# Molecular Taxonomy of Phytopathogenic Fungi: A Case Study in *Peronospora*

**DOI:** 10.1371/journal.pone.0006319

**Published:** 2009-07-29

**Authors:** Markus Göker, Gema García-Blázquez, Hermann Voglmayr, M. Teresa Tellería, María P. Martín

**Affiliations:** 1 Organismic Botany, Eberhard Karls University of Tübingen, Tübingen, Germany; 2 Departamento de Micología, Real Jardín Botánico, CSIC, Madrid, Spain; 3 Department of Systematic and Evolutionary Botany, Faculty Centre of Biodiversity, University of Vienna, Wien, Austria; Duke University Medical Center, United States of America

## Abstract

**Background:**

Inappropriate taxon definitions may have severe consequences in many areas. For instance, biologically sensible species delimitation of plant pathogens is crucial for measures such as plant protection or biological control and for comparative studies involving model organisms. However, delimiting species is challenging in the case of organisms for which often only molecular data are available, such as prokaryotes, fungi, and many unicellular eukaryotes. Even in the case of organisms with well-established morphological characteristics, molecular taxonomy is often necessary to emend current taxonomic concepts and to analyze DNA sequences directly sampled from the environment. Typically, for this purpose clustering approaches to delineate molecular operational taxonomic units have been applied using arbitrary choices regarding the distance threshold values, and the clustering algorithms.

**Methodology:**

Here, we report on a clustering optimization method to establish a molecular taxonomy of *Peronospora* based on ITS nrDNA sequences. *Peronospora* is the largest genus within the downy mildews, which are obligate parasites of higher plants, and includes various economically important pathogens. The method determines the distance function and clustering setting that result in an optimal agreement with selected reference data. Optimization was based on both taxonomy-based and host-based reference information, yielding the same outcome. Resampling and permutation methods indicate that the method is robust regarding taxon sampling and errors in the reference data. Tests with newly obtained ITS sequences demonstrate the use of the re-classified dataset in molecular identification of downy mildews.

**Conclusions:**

A corrected taxonomy is provided for all *Peronospora* ITS sequences contained in public databases. Clustering optimization appears to be broadly applicable in automated, sequence-based taxonomy. The method connects traditional and modern taxonomic disciplines by specifically addressing the issue of how to optimally account for both traditional species concepts and genetic divergence.

## Introduction

A reliable taxonomy is crucial for the assessment of biodiversity and for the categorization of habitats based on their species composition, and is crucial for comparative studies involving model organisms. In addition, species definitions for pathogens have considerable practical impact for protective measures and for biological control. However, delimiting taxa is challenging in the case of organisms for which (almost) exclusively molecular data are available, even in the case where robust phylogenetic hypotheses can be inferred. For microorganisms such as prokaryotes, fungi, and many other unicellular eukaryotes, only few diagnostic characters may be present, and an increasing number of such organisms are only known by their DNA sequences [1]–[10]. Frequently, molecular taxonomy is necessary to validate established species concepts and identify those that require a taxonomic revision even if phenotypic and ecological characteristics are well-established (e.g. highly specialized parasites). Molecular data are also essential to detect so-called cryptic (or pseudocryptic) species [11], i.e. species for which no morphological differences exist (or have not been determined so far), and to analyze sequences that have been directly sampled from their natural environment, e.g., in the context of metagenomics projects [12], [13], and for the early detection of pathogens in plant material to initiate quarantine measures [14], [15]. Despite its obvious utility in a number of cases, the entire concept of molecular taxonomy has been intensively debated in the literature, particularly regarding barcoding [16]–[18].

For molecular species delimitation, researchers mostly use a predefined threshold *T* for pairwise genetic distances in clustering algorithms to assign sequences to molecular operational taxonomic units [1]–[3], [5], [8]–[10]. Values of *T* used for clustering differ in the literature, even if applied to the same groups of organisms and molecular markers [4], [6], [7], [10] as they are often selected arbitrarily or based on a tradition that emerged in recent years for the sake of comparability between studies [19]–[21]. However, diversity estimates (including the total number of species on earth) are strongly dependent on T (e.g. [22]). Moreover, the clustering algorithm used, which is also crucial for the content and the shape of the clusters formed [23: 192], has hardly been addressed. Even in the context of linkage clustering, one can vary between the extremes of single linkage and complete linkage (see overviews in, e.g., [23], [24]). The differences in mean and maximum within-cluster distances, for a given *T*, may be much more pronounced between clusters if single-linkage clustering is applied [23: 192], which is important because morphologically defined lineages may display distinct genetic divergence [25]. Methods more advanced than linkage clustering have been suggested [26], [27], [28], but these are designed for identification, i.e. the assignment of query sequences to predefined groups, and thus require a correct reference taxonomy. However, even in the case of organisms with well-established microscopical characteristics, misidentifications are frequent, and sequences in public databases can be mislabelled. Conversely, algorithms based on coalescent theory can be used to estimate species boundaries (e.g. [29]), but, among other intricacies, these rely on multi-locus sequencing [30:491] and can hardly be applied to environmental samples and to data that are only available as accessions in public databases. With regard to the intense debate between molecular taxonomists and traditional morphologists, particularly in the context of DNA barcoding [16], [18], it is becoming obvious that methods are useful that can maximize the agreement between molecular and traditional taxonomy. Such methods would require that a set of specimens identified using traditional techniques serve as the reference points, but should not require that the identifications are entirely correct.

We here use *Peronospora* Corda and *Pseudoperonospora* Rostovzev (Peronosporales, Peronosporomycetes) internal transcribed spacer nuclear ribosomal DNA (ITS nrDNA) sequences as a model system. *Peronospora* is the most species-rich genus within the downy mildews, which are obligate plant pathogens mostly infecting dicots [31]. In this group, taxonomically useful morphological characters are few. The delimitation of many, but not all, species by morphometric methods is still an imprecise activity owing both to the great influence of the environment on the morphology of most somatic structures and also to the lack of technical advances [32]. These difficulties are reflected by the history of *Peronospora* taxonomy. De Bary [33] applied a broad species concept in which usually all *Peronospora* samples infecting a specific host family were considered as a single species. This concept was challenged by authors such as Gäumann [34], [35], Gustavsson [36], [37] and Săvulescu [38], who assumed much narrower species boundaries and were in turn challenged by Yerkes and Shaw [39].

Currently, more than 400 ITS nrDNA fragments of *Peronospora* and its sister genus, *Pseudoperonospora*, are stored in public databases (NCBI/GenBank, EMBL, DDBJ). To date, these data represent the most comprehensively sampled marker for the downy mildews; other genes are much less studied. The systematic and taxonomic potential of the ITS rDNA has often been acknowledged in the case of these organisms. In particular, the ITS has been reported to be in accordance with the affiliation to plant hosts: in the vast majority of cases, concepts based on the assumption of high host specificity and, thus, narrow species boundaries as put forward by authors such as Gäumann [34], [35] and Gustavsson [36], [37] have been found almost always in agreement with clades supported by ITS nrDNA data in the downy mildew genera *Peronospora* [40], [41], *Hyaloperonospora* [42]–[44], *Plasmopara* [45], [46], and *Bremia* [45]. As a rule with few exceptions (e.g. [47]), downy mildew (DM) species infect only a single host species or several host species within the same genus. Thus, Göker et al. [44] concluded that a combination of host and molecular characters is sufficient to solve the species problem in downy mildews. It is thus of interest whether the proposed optimization of sequence clustering using the host species as reference data can be applied to address this problem, and whether its outcome is similar to that of taxonomy-based optimization.

We here apply non-hierarchical clustering to the *Peronospora* and *Pseudoperonospora* ITS nrDNA data matrix to obtain taxonomic units (TU) that are in optimal agreement with the currently accepted taxonomy. To determine the best clustering parameters *T* and *F*, we automatically obtain a representation of the alleged affiliation of sequences to taxa by extracting the taxonomy from the *Peronospora* and *Pseudoperonospora* ITS GenBank entries and by filtering out incorrectly formatted taxon names. An alternative reference dataset represents the information on the plant hosts as far as provided in the public ITS sequences. The optimization method is also used to compare distinct sequence alignment programs and distance functions. Resampling and permutation techniques are used to study the robustness of the optimization regarding taxon sampling and errors in the reference partition. The taxonomic units, which may serve as our best estimates for species in future studies, are assigned to informal, or, as far as possible, formally defined taxon names from literature. Accordingly, we provide a corrected nomenclature for all current GenBank ITS sequences of *Peronospora* and *Pseudoperonospora*. Newly obtained sequences from Chenopodiaceae hosts are used as an example for molecular identification based on the corrected nomenclature. The outcome is discussed regarding current concepts about “species” within downy mildews and the general applicability of our methods for automated, sequence-based taxonomy.

## Materials and Methods

### Sample sources and DNA extraction

ITS nrDNA sequences of *Peronospora* and *Pseudoperonospora* were downloaded on 22/10/2008 from the NCBI/GenBank database using its taxonomy query portal. The query was chosen so as to obtain sequences comprising ITS1, 5.8S and ITS2 nrDNA. According to recent molecular phylogenetic studies [41], [48], [49], *Pseudoperonospora* is the sister genus of *Peronospora* and thus was included as the outgroup for rooting the trees. Sequences and information on taxa and hosts to define the reference partitions (see below) were extracted from the complete GenBank flat files using the program gbk2fas (freely available at http://www.goeker.org/mg/clustering/). A small number of sequences (12) were shorter than 750 bp and were removed prior to phylogenetic and clustering analysis in order to restrict the dataset to accessions comprising (almost) full-length ITS1 and ITS2 segments.

We sequenced 14 additional isolates to use as test queries; voucher information is listed in Table 1. For DNA extraction of infected herbarium, host tissue specimens, the E.Z.N.A. Fungi DNA Miniprep Kit (Omega Biotech) was used according to the manufacturer's protocol. ITS1-O (5′-CGG AAG GAT CAT TAC CAC) [40] and ITS4-H [44], a modification of ITS4 [50] were used as PCR and cycle-sequencing primers. In some cases, a nested PCR approach had to be used in which ITS5 [50] and ITS4-H were used in the first PCR and ITS1-O was combined with ITS4-H in the second PCR. PCR was carried out with Ready-to-Go-PCR Beads (GE Healthcare Life Sciences) in a MJ Research-PTC-200 thermocycler; settings were as in [51]. The PCR products were purified using QIAquick (QIAGEN, Valencia, California, USA) and sent to Secugen S. L. (CIB, Madrid) for sequencing.

**Table 1:** GenBank Accession Numbers and Voucher Information for Query Sequences.

Host	Geographical origin, source or herbarium number	*Peronospora* species (according to host and morphology)	GenBank accession no.	Closest neighbours in clustered dataset	Distance to closest neighbours
*Atriplex hortensis*	Spain, Asturias, Carcabada, MA-Fungi 27736	*Pe. minor*	FM863725	DQ643842 (TU 5)	0.000000
*Chenopodium album*	Spain, Burgos, Cornudilla, MA-Fungi 27855	*Pe. variabilis*	FM863718	EU113303, EU113304, EU113305, EU113306, EU113307, EU113308, EU113310 (TU 49)	0.000000
*Chenopodium album*	Spain, Gerona, Bolvir, MA-Fungi 27858	*Pe. variabilis*	FM863720	EU113303, EU113304, EU113310 (TU 49)	0.000000
*Chenopodium album*	Spain, Gerona, Campdevànol, MA-Fungi 27857	*Pe. variabilis*	FM863723	EU113309 (TU 49)	0.000000
*Chenopodium album*	Spain, Gerona, Isóvol, MA-Fungi 27859	*Pe. variabilis*	FM863716	EU113303, EU113304, EU113309, EU113310 (TU 49)	0.000000
*Chenopodium album*	Spain, Gerona, Puigcerdà, MA-Fungi 27854	*Pe. variabilis*	FM863719	EU113309 (TU 49)	0.000000
*Chenopodium album*	Spain, Huesca, Canfranc, MA-Fungi 27861	*Pe. variabilis*	FM863717	EU113303, EU113304, EU113309, EU113310 (TU 49)	0.000000
*Chenopodium album*	Spain, La Rioja, Ansejo, MA-Fungi 27862	*Pe. variabilis*	FM863721	EU113303, EU113304, EU113305, EU113306, EU113307, EU113308, EU113310 (TU 49)	0.000000
*Chenopodium album*	Spain, Lerida, Esterri d'Àneu, MA-Fungi 27856	*Pe. variabilis*	FM863724	AF528556, AF528557, AY211017, EF614959, EF614961, EF614962, EF614963, EF614965, EF614966, EF614967, EF614968 (TU 49)	0.000000
*Chenopodium album*	Spain, Lérida, Las Bordas, MA-Fungi 27864	*Pe. variabilis*	FM863722	EU113310, EU113304, EU113303 (TU 49)	0.000000
*Chenopodium bonus-henricus*	Spain, Asturias, Leitariegos, MA-Fungi 27850	*Pe. boni-henrici*	FM863712	AY198286, EF614952, EF614953 (TU 73)	0.001294
*Chenopodium bonus-henricus*	Spain, Huesca, Baños de Benasque, MA-Fungi 27849	*Pe. boni-henrici*	FM863713	AY198286, EF614952, EF614953, EF614954 (TU 73)	0.000000
*Chenopodium bonus-henricus*	Spain, Huesca, Baños de Panticosa, MA-Fungi 27847	*Pe. boni-henrici*	FM863715	AY198286, EF614952, EF614953, EF614954 (TU 73)	0.000000
*Chenopodium bonus-henricus*	Spain, Huesca, Baños de Panticosa, MA-Fungi 27848	*Pe. boni-henrici*	FM863714	AY198286, EF614952, EF614953, EF614954 (TU 73)	0.000000

Collection data (columns 1–3), GenBank accession numbers and molecular taxonomic results (columns 5–6) for the sequences newly obtained in the course of this study. The material is preserved in public collections: MA-Fungi, Real Jardín Botánico de Madrid, Spain and LISE, Estaçao Agronomica Nacional, Portugal.

### Reference data for optimization

Clustering optimization is based on one to several reference *partitions*. In contrast to, e.g., a phylogenetic tree, a partition is non-nested data structure in which each object (here: each sequence) is assigned to exactly one cluster. The affiliation of specimens to taxa, for example, represents a partition, if the taxa are of the same taxonomic rank. The clustering parameters are optimized so as to obtain the highest agreement between the partition inferred by clustering and the reference partition(s). The first reference partition consisted of the taxonomic affiliations of the corresponding specimens as defined in the GenBank flat files in the “organism” descriptor. The NCBI taxonomy of *Peronospora* and *Pseudoperonospora* does not always contain validly published species names; for instance, organism entries such as “*Peronospora* sp. SMK20063” are present. These accessions were removed prior to taxonomy-based clustering optimization. Names such as “*Peronospora farinosa* f. sp. *chenopodii*” were reduced to their species binomial (“*Peronospora farinose*” in that case).

A second reference partition was constructed from the host names as far as included in the GenBank entries. Host names such as “*Cucumis melo* var. *reticulatus*” were reduced to their species binomial (“*Cucumis melo*” in our example); in contrast to the use of the *Peronospora* taxonomy, names such as “*Atriplex* sp.” were retained. Cross-comparison of host names and searching for these names in the NCBI taxonomy revealed a number of typing errors, which were corrected prior to clustering optimization. Accessions lacking host information were removed prior to host-based clustering optimization. Importantly, the processing of both the organism entries and the host names could be partially (correction of typing errors in host names) or even fully (all other procedures) automated; e.g. extracting species binomials from organism entries was based on regular expressions. All corrections are documented in the supporting material (supporting file S2).

### Distance calculation

Sequences were aligned using poa [52] in default mode (see [40] regarding the choice of the alignment software). Pairwise uncorrected (“P”) distances (treating gaps as missing data) were inferred with PAUP* version 4b10 [53], which were used for taxonomy-based clustering optimization after removal of accessions with taxonomically invalid names and used for host-based optimization after removal of accessions lacking interpretable host names (see above). To assess the effect of alternative DNA alignment software and/or more complex distance functions on the clustering results, alignments were inferred with four other software packages and additional distance matrices with PAUP* and RAxML version 7.04 [54]; detailed information is found in supporting file S3. A total of 108 distinct distance matrices were subjected to clustering optimization, and it was reported whether a significantly better result than the main analysis based on the fast poa alignment and simple uncorrected distances was obtained.

### Clustering optimization

To define taxonomic units as the basis of an objective classification incorporating evidence from traditional taxonomy, we followed a two-step approach: (1) apply a specifically parametrized clustering algorithm to infer a non-nested classification (i.e., a partition; see above) from the ITS sequences; (2) determine the agreement between this partition and the reference partition. If the steps (1) and (2) are repeated for a range of clustering parameters, the best parameters, globally, are those for which the agreement with the reference partition is highest. We here use either (2a) the species affiliations extracted from the current GenBank classification system for *Peronospora* and *Pseudoperonospora* or (2b) the host taxa of the two genera as reference partition.

Step 1 relies on a non-hierarchical clustering algorithm, i.e. a method that outputs a non-nested classification (partition) of the objects [55], [24: 358]. Here, we apply non-hierarchical linkage clustering, which is based on a fixed distance threshold *T* and the notion of a “link” between two objects. A link exists between the two objects, if the distance between them is equal to or lower than *T*. The algorithm starts by assigning each object to a cluster of its own and then checks whether fusing is necessary, beginning with the smallest distances up to *T*. The linkage fraction *F*, i.e. the proportion of links present between the objects in two distinct clusters (relative to the total number between-cluster distances), determines whether the two clusters are fused. Non-hierarchical *single*-linkage clustering [55] fuses two clusters if a single link exists between the two objects. Technically, *F* is then equal to 0.0 because apart from the single link no further links are required. On the contrary, non-hierarchical *complete*-linkage clustering [24: 358] fuses two clusters only if the distances between all the objects in the clusters are links. Here, *F* is equal to 1.0. A full range of clustering techniques intermediate between the two extremes can be applied by using values for *F* between 0.0 (single linkage) and 1.0 (complete linkage), and the globally optimal combination of *T* and *F* can be determined. *F* has hardly been addressed in the recent literature on molecular taxonomy. The clustering of triplets of sequences, for example, has been regarded as “logically inconsistent” if only two of the three distances represent links [56]. However, for a given *T*, mean and maximum within-cluster distances can vary much more between clusters for small values of *F* [23: 192].

The agreement with the reference partition (step 2) is evaluated using a rescaled version of the Rand index [57] (called modified Rand index or MRI; [58], formula 5). The Rand index relates the number of pairs of objects *i* and *j* which are either in the same group in the partitions *a* and *b* or in different groups in both partitions to the total number of such pairs. Let *g_ij_* be an accessory function that returns 1 if a cluster that contains *i* and *j* exists in partition *a* and in partition *b*, returns 1 if such a cluster neither exists in *a* nor in *b*, and returns 0 in all other cases. The Rand Index is then defined as
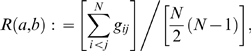
(1)where *N* corresponds to the total number of objects and, thus, the denominator to the number of pairs of (non-identical) objects. The MRI is based on the general formula of an index corrected for chance:
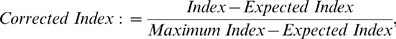
(2)where “Index” is the numerator of equation 1, the maximum index is assumed to be 1.0 and the formula for the expected index is the one derived by [58]. Hence, the MRI includes a correction for the effect of coincidence, i.e. it is maximal (1.0) in the case of identical partitions but obtains values around 0.0 in the case of random partitions.

Given a reference partition, the optimal clustering parameters are those that yield the highest MRI among all candidate parameter combinations. Values of *T* and *F* were varied between 0.0 and 1.0, with a step width of 0.0001 (*T*) or 0.05 (*F*). In the case of ties, the median of the optimal values was recorded. To visualize the results, cluster affiliations from the optimal clustering were mapped on the phylogenetic tree inferred as described below. The clustering optimization procedure is included in the program optsil freely available at http://www.goeker.org/mg/clustering/.

### Assessing robustness of clustering optimization

An important feature of the optimization process is whether it is robust regarding taxon sampling. That is, the inferred optimal F and T values should remain optimal even if the underlying set of sequences is modified, e.g., by including additional samples. To assess the stability of our clustering optimization strategy, we applied taxon jackknifing [59]. Within each jackknife replicate, a defined proportion of the sequences was selected at random and removed before optimizing the parameters, ranging from 5% to 50% of the sequences, and using a step width of 5%. For each removal setting, 1,000 taxon jackknife replicates were conducted, and the range of optimal clustering parameters was reported for each replicate. Taxon jackknifing is also implemented in the optsil program; we here applied it only to the uncorrected distances inferred from the poa alignment.

In theory, each resulting cluster defines a taxonomic unit equalling a *Peronospora* or *Pseudoperonospora* species. However, because of limitations in distance calculation, considerable difference in genetic divergence between the species, sequencing artefacts, or misidentification or mislabelling of specimens, even the optimal MRI may not achieve 1.0 (as shown below, the mislabelling problem is the most apparent in the dataset, whereas clustering of *Peronospora* and *Pseudoperonospora* ITS rDNA sequences works very well). Like standard statistical optimization procedures such as ordinary least-squares regression, maximum parsimony or maximum likelihood phylogenetic inference, which do not assume that the global optimum can actually be obtained [60], clustering optimization does not presuppose that full agreement between partitions can be obtained and that the reference partition is completely error-free (see above). On the other hand, it is of interest how robust the procedure is against the proportion of errors in the reference partition for the dataset under study. To assess this effect, we introduced a defined proportion of errors (between 5% and 50%, step width was 5%) in the reference partition before optimizing the parameters. Errors to be introduced were selected at random in each of the 1,000 replicates per proportion by swapping the affiliations of two randomly selected sequences until the requested proportion of errors was achieved. As above, the range of optimal clustering parameters was reported for each replicate, and the analysis was restricted to the uncorrected distances inferred from the poa alignment.

### Phylogenetic analysis

A phylogenetic tree, even if well-resolved, is compatible with a (potentially large) number of non-nested classifications and does not directly indicate taxon boundaries. Accordingly, inferring a tree cannot be used as a substitute of a non-hierarchical clustering approach to define taxonomic units. However, it is of interest whether the clusters obtained are monophyletic in a tree because well-supported branches may disagree with a classification. In other words, that a cluster is not supported as non-monophyletic in a tree (i.e., that it is either supported as monophyletic or that there is no support for its status whatsoever) is a necessary, but not a sufficient condition for accepting the cluster as a taxon. Importantly, a lower pair-wise distance does not always indicate a closer phylogenetic relationship [30: 165]. This issue has lead to the wide-spread avoidance of UPGMA [61] (a hierarchical clustering algorithm) in phylogenetic studies.

We thus inferred phylogenetic trees from the poa alignment under the maximum likelihood criterion with RAxML version 7.04 [54]. To establish node support, we used RAxML's novel fast bootstrap algorithm [62] with 100 replicates and subsequent search for the globally best maximum-likelihood tree in conjunction with the GTRMIX model approximation [63] (command-line switches -m GTRMIX -f a -# 100). To assess the impact of sequence alignment, RAxML trees were also inferred under the same settings from the other alignments, an approach known as multiple analysis [64], [65]; for details, see supporting file S3. Bootstrap support was also calculated with PAUP* version 4b10 [50] with 1,000 replicates under the maximum parsimony criterion [66] after exclusion of parsimony-uninformative sites. Per replicate, 10 independent random sequence addition runs followed by TBR branch swapping were conducted, saving only a single most parsimonious tree per run.

### Improved classification of GenBank Peronospora ITS sequences

The optimal clustering parameters were applied to the full GenBank dataset, and the resulting clusters were compared with the organism entries of the respective accessions to screen for discrepancies. Host information from GenBank was also taken into account; if necessary, the original literature was checked for the possibility to resolve additional conflicts. Literature references could also be extracted from the GenBank flat files for most accessions and are contained in supporting file S2. Basically, two types of discrepancies can occur between clusters and reference taxa: (I) A given reference taxon can be distributed among several clusters, or (II) a given cluster can comprise more than one reference taxon.

In the case of *Peronospora*/*Pseudoperonospora*, there are several possible causes for (I). Firstly, the taxon (here: species) may occur on several host species or even genera, which correspond to the clusters (Ia); this implies that the discrepancy is easy to interpret biologically, i.e., host specificity is higher than reflected in the current taxonomy. If the taxon is monophyletic in the tree anyway, a taxonomic revision is only needed to solve a *ranking* problem and, hence, not as urgent as if the taxon is non-monophyletic in the tree and a taxonomic revision needs to solve a grouping problem. In the latter case, only molecular tools can be used to distinguish the species in the lack of morphological differences. Secondly, it may be that the species does not need revision, but that its ITS sequences are considerably more variable than the majority of other *Peronospora* and *Pseudoperonospora* species. A prerequisite for this condition is that the taxon is monophyletic and the clusters cannot be interpreted regarding differences in host specificity. The high variability may be caused by sequencing (or alignment) artefacts (Ib) or truly reflect the species' genetic diversity (Ic). Obviously, only Ic presents a conceptual problem for the clustering optimization approach, whereas Ib presents practical problems for identification. While Ib and Ic may be hard to distinguish in many cases, we used a workaround and determined the number of ambiguous base calls within each sequence as a rough measure for sequence quality.

Regarding discrepancy type II, we have to distinguish between cases in which the distinct taxa within the cluster occur on the same host species or at least on the same host genus (IIa), and cases in which they occur on distinct hosts (IIb). Ambiguous cases caused by doubtful host taxonomy (“(IIa)”) cannot be avoided, and we thus confine IIb to the occurrence on distinct host families. IIa indicates that the discrepancy is most likely caused by a naming problem, e.g. by the use of synonyms, or because the current classification overestimates the diversity in these cases. While condition IIb may be an artefact caused by mislabelled GenBank accessions, it is also known that some *Pseudoperonospora* species are not particularly host-specific [47], and all in all the clusters would be less easy to interpret biologically if IIb were a frequent condition. Alternatively, IIb could also be caused by significantly less sequence variability within some taxonomic groups compared to the others, then failing to distinguish even highly host specific entities using the ITS region. Unless otherwise indicated, our notes on the host taxonomy (also included in supporting file S2) below follow the release of the GenBank/NCBI taxonomy release of 19/01/2008.

Accordingly, a main hypothesis of our study is that Ia is much more frequent than Ib-Ic and that IIa is much more frequent than IIb. A confirmation of this hypothesis implies that a combination of molecular and ecological information (i.e., host data) is likely to solve the major problems of *Peronospora* and *Pseudoperonospora* taxonomy in the near future. We test this hypothesis by using both the hosts and the GenBank taxonomy for clustering optimization, by comparing the results, and by determining the relative number of occurrences of the main types of discrepancies.

The splitting of taxa with a valid taxon name causes nomenclatural problems because only one of the resulting new groups can be assigned to the previously used name. Fusion of taxa with valid taxon names causes nomenclatural problems because only one of the names can be assigned to the resulting group. Also, type hosts are lacking for most of the species descriptions in the monograph of Gäumann [35] if they are based on collections from more than one host species. We thus report the type host or alternatively a list of authentic hosts for all of the valid taxon names in the sequence set, and provide the arguments for our naming decisions in the supporting material (supporting file S2). Regrettably, we have no experimental access to many of the GenBank accessions, and a taxonomic revision of the two genera is far beyond the scope of the present study. However, we provide valid taxon names if possible and provisional informal names otherwise for all of the clusters in our dataset. In many cases, molecular taxonomy enables us to recognize taxon names that are based on outdated, broad species concepts, and to suggest an improved nomenclature. These give important hints for further studies on *Peronospora* and *Pseudoperonospora* taxonomy and can be used for identification purposes until valid taxon names have been established.

### Molecular identification of *Peronospora*

In order to test the suitability of the clustering optimization for the identification of *Peronospora* samples, we added the 14 newly sequenced downy mildew samples to the previously generated multiple sequence alignment, using the -read_msa option of the poa alignment software. Distances were calculated with PAUP* as described above and the closest neighbours of each query sequence, i.e. the least distant of the previously classified sequences, were determined and recorded together with their affiliation to a TU as previously obtained by applying the optimized clustering parameters.

## Results

### Taxonomic units based on clustering optimization

**Figure 1:**
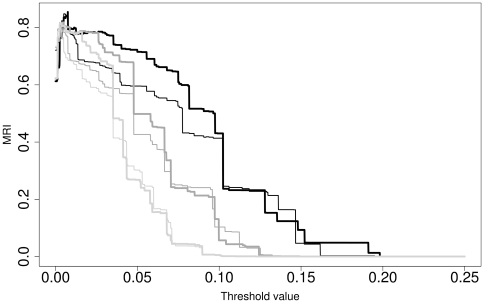
Optimization plots. Modified Rand Index (MRI) plot based on the poa alignment, uncorrected distances, the globally optimal *F* value (1.0) and two suboptimal *F* values (0.0 and 0.5). Axes: x-axis, *T* values examined (values larger than 0.25 gave the same result because all sequences were assigned to a single cluster); y-axis, resulting MRI values for taxonomy-based optimization (thick lines) and host-based optimization (thin lines). Colours: black, *F* = 1.0; dark grey, *F* = 0.5; light grey, *F* = 0.0.

**Figure 2:**
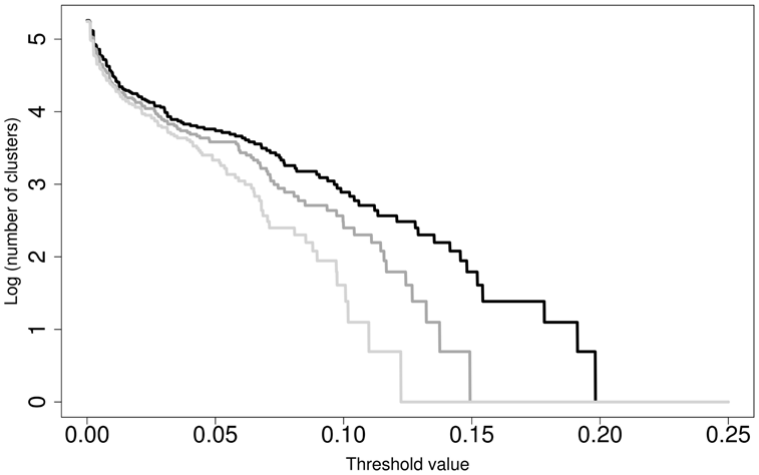
Dependency of the number of molecular taxonomic units (TU) on T and F. The subset of the data with correctly formatted taxon names was analysed. Axes: x-axis, *T* values examined (values larger than 0.25 gave the same result because all sequences were assigned to a single cluster); y-axis, natural logarithm of the resulting number of clusters (TU) for three selected values of *F*. Colours: black, *F* = 1.0; dark grey, *F* = 0.5; light grey, *F* = 0.0.

The complete sequence dataset downloaded from GenBank included 439 ITS nrDNA sequences, 427 of which were sufficiently long (see above). Within the latter, 354 accessions contained a correctly formatted “organism” entry, and 388 contained a correctly formatted host name. The reference partition constructed from *Peronospora*/*Pseudoperonospora* species names comprised 86 distinct entries, the one constructed from the hosts comprised 141 distinct entries (from 72 distinct genera). The poa alignment had a length of 2118 bp, which was partly caused by some sequences comprising parts of the small subunit rDNA and by the long ITS1 insertions in the *Trifolium* parasites [40], [41]. Taxonomy-based optimization of the P distances inferred from the poa alignment resulted in an optimal modified Rand Index (MRI) value of 0.85485, corresponding to *F* = 1.0 and *T* = 0.0075. In host-based optimization, the best MRI value was 0.85204, which was obtained if *exactly the same F* and *T* values were used for clustering. The optimization plot for the poa alignment, *F* = 1.0 and both reference partitions are shown in Fig. 1. Plots for two suboptimal *F* values, 0.0 and 0.5, are also shown. If applied to the full alignment of 427 sequences, the optimal clustering parameters resulted in 117 clusters (taxonomic units or TU) and 199 distinct combinations of TU and GenBank “organism” entry. The effect of T and F on the resulting number of clusters (TU) is shown in Fig. 2. Twenty distinct “organism” entries appeared in more than one TU, whereas 23 TU where associated with more than one “organism” (supporting file S2). The best MRI values obtained for the poa alignment and all distance formulae, dependent on the tested *F* values, is shown in supporting file S3. While an additional local maximum is present in the case of taxonomy-based optimization for *F* = 0.25 and *F* = 0.30, F = 1.0 gives far superior MRI values than any other *F* value for both partitions. Using other alignment programs and/or distance formulae did not result in considerably higher MRI values; rather, improvements were restricted to the third position after the decimal point. All alignments, selected distance matrices and the original optimization results for all of them are included in supporting file S1.

### Robustness of clustering optimization

**Figure 3:**
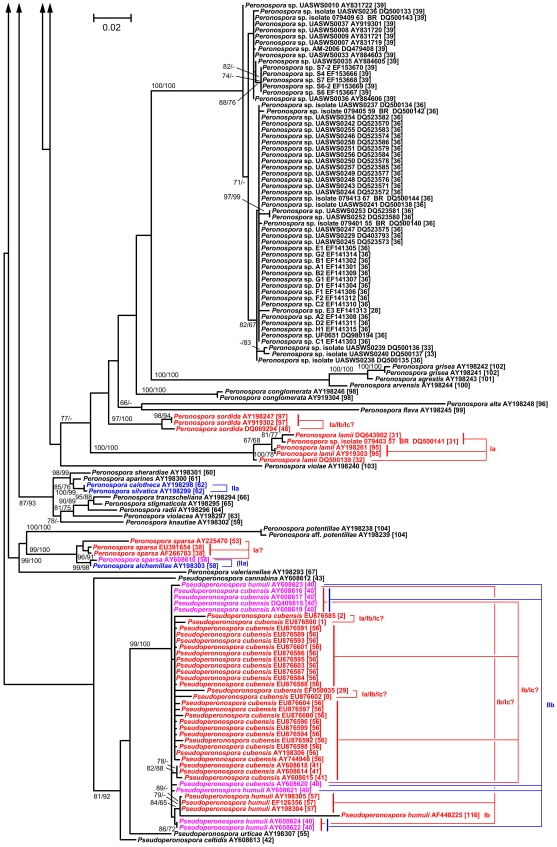
Maximum-likelihood tree, bottom part. Phylogram as inferred with RAxML and rooted with the *Pseudoperonospora* sequences present in the dataset. Branches are scaled in terms of the number of substitutions per site. Numbers above/below the branches are maximum likelihood and maximum parsimony bootstrap support values from 100 replicates. The sequence labels contain the “organism” entry and the accession number from the GenBank files; for the validity of these entries, the corrected “organism” names and the revised taxonomy, see supporting file S2. Taxonomic unit (TU) numbers from optimal clustering settings are provided in rectangular brackets. These numbers are only used to circumscribe the TU; they do not indicate relationships between the TU (e.g. TU 16 is not closer to TU 15 than to TU 91). Red labels denote accessions affected by type I conflicts, blue labels by type II conflicts, mauve labels by both type I and II conflicts and green labels by database errors due to incorrect data submission. The red (type I) or blue (type II) lines connect the accessions affected by the respective conflict, with the conflict subtype given to the right. Type I concern the presence of the same taxon in different clusters (TU), type II the presence of several taxa within the same cluster (TU). Subtypes: Ia, different TU correspond to different hosts; Ib-Ic, different TU correspond to the same host; Ib, different TU are effected by sequencing/alignment artefacts; Ic different TU are effected by high genetic variability; IIa different taxa within a TU occur on the same host species/genus; (IIa) different taxa within a TU occur on different host genera within the same family; IIb different taxa within a TU occur on different host families. The tree is continued in Fig. 4.

Results from taxon jackknifing and from random permutations of the reference partition are shown in supporting file S4. In jackknife analysis of the taxonomy-based optimization, the optimal *F* values inferred from the complete dataset were also optimal in almost all replicates if up to 10% and still in the majority of the replicates if up to 25% of the sequences are deleted (Figs. 1–2 in supporting file S4), while the original optimal *T* value is optimal in the majority of cases for up to 10% deletion, whereas almost always lower *T* values were preferred for higher proportions (Figs. 3–4 in supporting file S4). In random permutation analysis of the taxonomy-based reference partition, the optimal *F* and *T* values inferred from the complete dataset are still optimal in almost all replicates if up to 20% and still in the majority of the replicates if up to 40% errors are introduced (Figs. 6–9 in supporting file S4).

In jackknife analysis of the host-based optimization, the optimal *F* values inferred from the complete dataset were also optimal in almost all replicates if up to 15% and still in the majority of the replicates if up to 40% of the sequences are deleted (Figs. 11–12 in supporting file S4), while *T* values at most as high, but almost always lower than the original optimal *T* value were preferred for all tested deletion proportions (Figs. 13–14 in supporting file S4). In random permutation analysis of the host-based reference partition, the optimal *F* value inferred from the complete dataset was still optimal in almost all replicates if up to 25% and still in the majority of the replicates if up to 50% errors are introduced (Figs. 16–17 in supporting file S4), while the original optimal T value was preferred for up to 5% errors in almost all replicates and up to 25% errors in the majority of the replicates; for higher deletion proportions, the best *T* value was almost always smaller (Figs. 18–19 in supporting file S4).

As expected, the medians of the MRI values remained stable with an increasing proportion of deleted sequences, whereas their range linearly increased (Figs. 5, 15 in supporting file S4); also, the medians of the MRI values linearly decreased with an increasing proportion of errors in the reference partition, whereas their range remained stable (Figs. 10, 20 in supporting file S4).

### Phylogenetic inference

**Figure 4 pone-0006319-g004:**
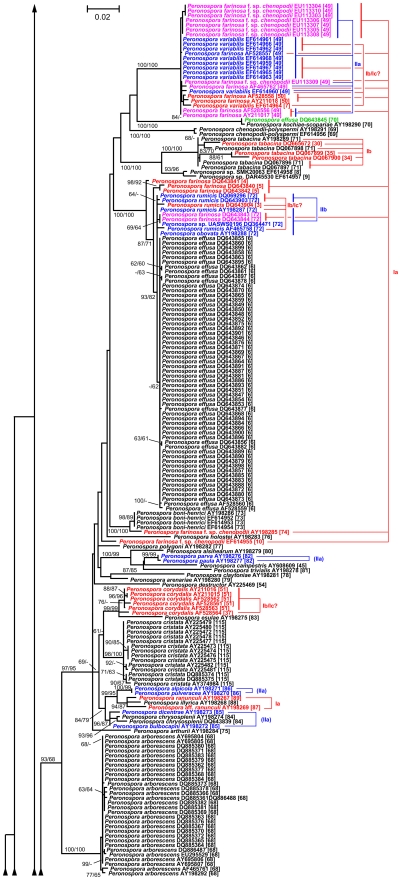
Maximum-likelihood tree, central part. Phylogram as inferred with RAxML; continuation of Fig. 3 (connections indicated by arrowheads). For a description of the sequence labels and the colouring, see legend to Fig. 3. The tree is continued in Fig. 5.

**Figure 5:**
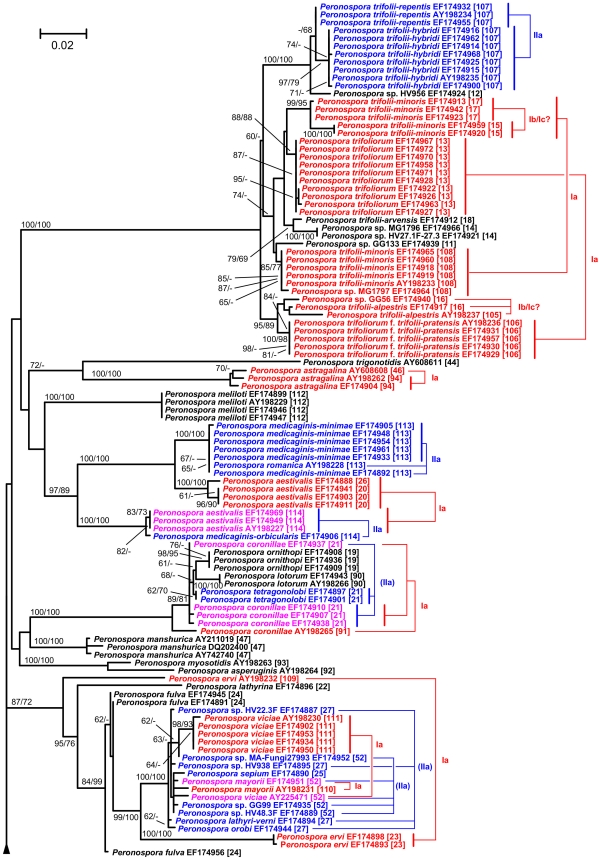
Maximum-likelihood tree, top part. Phylogram as inferred with RAxML; continuation of Fig. 4 (connections indicated by arrowheads). For a description of the sequence labels and the colouring, see legend to Fig. 3.

The maximum-likelihood tree inferred from the poa alignment had a log likelihood of -16392.00 and is shown in Figs. 3, 4, 5, together with the numbers of the taxonomic units obtained by clustering the 427 sequences using the optimal parameter settings. In a previous comprehensive study on *Peronospora* phylogeny [41], backbone resolution of the phylogenetic trees was relatively low. The poa maximum-likelihood tree showed the same pattern, even though the separation of *Peronospora* and *Pseudoperonospora* was well supported (Fig. 3). However, strong (93% under maximum likelihood, 68% under maximum parsimony) support was present for a large clade comprising mainly parasites of Caryophyllales and Ranunculales; a subclade of it comprising the same species except *Peronospora arborescens* was supported with 97% and 95%, respectively (Fig. 4). Some smaller groups with uniform host relationships are also well supported, e.g. a clade comprising four accessions of Rubiaceae parasites (98/99% bootstrap; Fig. 3). In contrast, a large monophylum of exclusively Fabaceae pathogens is present in the tree, but without support (Fig. 5). On the other hand, the tree contains a large number of near-terminal nodes that receive high support, most of which are equivalent to a taxonomic unit (Figs. 3–5). Even though not all taxonomic units are monophyletic in the tree, no taxonomic unit was found that conflicted with a well supported branch.

As for clustering optimization (see above), using other alignment programs than poa did not have a significant impact on the tree topologies and the support values. For instance, the large Fabaceae clade was not supported in any of the analyses, whereas the clade comprising mainly parasites of Caryophyllales and Ranunculales was well supported (93–98%) in analyses based on five of the six alignments. These alternative trees are described in detail in supporting file S3.

### Examples for taxonomic units and their interpretation

Our clustering-based taxonomic interpretation of the molecular taxonomic units (TU) are included in supporting file S2. Many taxonomic units were found that exactly correspond to *Peronospora* and *Pseudoperonospora* species names provided as Genbank “organism” identifiers. Full agreement is not restricted to taxa represented by single-sequences; for instance, the 30 *Pe. arborescens* sequences are placed solely and exclusively in TU 68, while the set of 14 *Pe. cristata* sequences exactly corresponds to TU 115 (see also [15]). Other taxonomic units are in full agreement with *Peronospora* species, but the GenBank annotation is erroneous. For instance, sequence DQ643845 is wrongly annotated as *Pe. effusa* on *Spinacia oleracea*, but has been collected on *Bassia scoparia*, as stated in the corresponding publication [67] and in accordance with its placement in TU 70 (*Pe. kochiae-scopariae*). The annotation errors and their corrections are listed in supporting file S2.

Discrepancy type Ia is also common in the dataset. For instance, *Peronospora aestivalis* is distributed over three TU (20, 26, 114), which exactly correspond to the infected species of *Medicago* hosts. This is also an example of nomenclatural intricacies because the authentic hosts of *Pe. aestivalis* include both *M. polymorpha* (TU 26) and *M. sativa* (TU 114). While a new species could be described without these difficulties on *M. truncatula* (TU 20), the situation is further complicated by the additional presence of *Pe. medicaginis-orbicularis* in TU 114, an example of discrepancy type IIa. Like *Pe. aestivalis*, *Pe. lamii* is split into several clusters (TU 31, 93, 95), which correspond to its plant host species. A third example for discrepancy type Ia is “*Peronospora farinose*”, which is distributed over seven clusters (TU 4, 5, 10, 49, 50, 72, 74) with distinct host species. However, this name is an example of an outdated, too broad species concept and more appropriate labels are already available in the literature such as *Pe. litoralis* and *Pe. minor* for accessions on *Atriplex* and *Pe. chenopodii*, *Pe. bohemica* and *Pe. variabilis* for samples from *Chenopodium* species (listed in detail in supporting file S2; see also [67], [68]). In addition, “*Peronospora farinosa*”, despite being widely used, currently represents a dubious name, as it was described from *Atriplex* sp. without further details, and no extant type specimen is known.

Discrepancies type Ib or Ic affected a number of taxonomic units but were hard to distinguish because the recorded number of ambiguous base calls is an insufficient indicator for erroneous sequences. Evident cases of sequencing errors (type Ib) are the highly aberrant AF448225 sequence (*Pseudoperonospora cubensis*) as well as the *Peronospora tabacina* sequences DQ067900 and DQ067899 with many ambiguous bases. While more discrepancies based on sequencing artefacts cannot be ruled out with certainty, it is likely that at least in some cases true genetic diversity is responsible for taxa to be distributed over several clusters. For instance, the two *Ps. cubensis* clusters TU 40 (comprising four *Humulus* and five Cucurbitaceae pathogens) and TU 56 (host information mostly unavailable) are separated but rather large and thus appear to represent real genetic distinctness.

Several cases of discrepancy type IIa are observed in the dataset. For instance, *Peronospora trifolii-repentis* and *Pe. trifolii-hybridi* are merged in TU 107, corresponding to our previous interpretation [40], as are *Pe. romanica* and *Pe. medicaginis-minimae* (TU 113), and *Pe. calotheca* and *Pe. silvatica* (TU 62), all of which occur on distinct species within the same host genus, respectively. A number of TU are composed of collections from host species within distinct, but closely related or even hard to separate (e.g. *Lathyrus* vs. *Vicia* host genera [69]. Examples are TU 21 on *Tetragonolobus* (now included in *Lotus*) and *Coronilla* (Fabaceae, Tribe Loteae), TU 27 on *Lathyrus* and *Vicia*, TU 52 on *Lathyrus*, *Pisum* and *Vicia* (Fabaceae, Tribe Fabeae; see also [40], [70]), TU 82 on *Cerastium* and *Stellaria* (Caryophyllaceae, Alsinoideae [71]) and TU 58 on *Alchemilla* and *Rosa* (Rosaceae, Rosoideae). Distinct genera within the same family are also parasitized by TU 85 (Fumariaceae) and TU 86 (Ranunculaceae). Apart from *Pseudoperonospora cubensis* and *Ps. humuli*, which were postulated to be conspecific already by Choi et al. [47], only a single cluster, TU 72 (which indicates that *Pe. obovata*, *Pe. schachtii* and potentially *Pe. rumicis* are conspecific) occurs on hosts from distinct families, i.e. Amaranthaceae, Caryophyllaceae, and Polygonaceae, but even these families belong to the same order (Caryophyllales). Several of these discrepancy types IIb need to be investigated in detail to reveal whether they are really conspecific.

### Molecular identification of *Peronospora*

The results of ITS-based molecular identification of our example query sequences are included in Table 1. The closest distances of each query sequence to one or several reference sequences were all well below the clustering threshold, and the closest reference sequences belonged to TU as expected regarding the host- and morphology-based species identification, i.e. TU 5 (*Peronospora minor*), TU 49 (*Pe. variabilis*) and TU 73 (*Pe. boni-henrici*).

## Discussion

### Clustering optimization for molecular taxonomy

Our application of the clustering optimization procedure as implemented in optsil has had a number of benefits for *Peronospora* and *Pseudoperonospora* molecular taxonomy; analogous benefits are to be expected with other groups of organisms and other sequence regions. First, clustering optimization enabled us to test alignment programs and distance functions and to identify the best approach(es). Because each approach was independently optimized, the differences between the respective final best MRI values could be attributed to the ability of the method to recover taxonomic relationships. Here, only few combinations other than poa as alignment program and uncorrected (“P”) distances as distance formula performed better, and only marginally so. Beyond its speed, a second advantage of poa is that the sequences are aligned in input order without iterative refinement; i.e. adding query sequences does not change the positions of the reference sequences relative to each other [52], and, hence, the distances between them. Obviously, clustering optimization can also be used to assess the relative performance of alignment and distance methods if applied to other organisms and sequence regions. Because genetic divergence may differ between morphologically defined lineages [25], it is important that distinct optimal settings can be determined for distinct groups of organisms. Certain molecular loci may be present difficulties in case the genetic diversity is significantly lower in a specific lineage than average, then failing to distinguish closely related but genetically isolated lineages. This, of course, is not a problem of the optimization method itself but of the sequence region used. However, also in that case the method is very helpful in recognising critical groups which are in need of additional taxonomic investigations, and the MRI may well be used to compare the suitability of distinct molecular loci sequenced from the same organisms for molecular taxonomy.

Second, the optsil algorithm results in genetically homogeneous clusters (particularly if high *F* values are optimal, as in the case of our *Peronospora*/*Pseudoperonospora* dataset; see [23: 192] in optimal agreement with the reference partition of interest, which appears superior to the use of predefined thresholds [1]–[5], [8], [10], as long as the reference is biologically meaningful. Optimization may not work with all datasets, but failure can be ruled out if the optimal MRI values are significantly larger than 0.0 and much closer to 1.0. Importantly, the reference partition will never be forced upon the genetic data; only the threshold and *F* values can be optimized, whereas the composition of the resulting clustering is ultimately defined by the molecular sequences. Additionally, taxon jackknifing and random permutation can be used to assess the robustness of parameter optimization regarding taxon sampling and errors in the reference partition, respectively. Thus, the algorithm can be applied to each combination of a reference partition and a distance matrix; the user just has to closely examine the results. Applied to *Peronospora*/*Pseudoperonospora* ITS sequences, the algorithm is robust against misidentifications and a taxonomy that only partially reflects natural relationships, most likely because full agreement with the reference partition is not required. The optimal parameters can be used for sequence identification (as demonstrated using the 14 query sequences) and for the recognition of new sequence types just by applying them to enlarged datasets.

Third, reference taxonomies can usually be generated with ease. Automated processing of taxonomic descriptors found in public databases is possible, as applied here to obtain correctly formatted taxon names according to the Linnéan nomenclature. Parameter optimization can then be conducted for the subset of the data characterised by proper species names, as done here when reducing the 427 GenBank accessions to the 354 used for taxonomy-based optimization. This approach is reasonable for all clades except very large ones which nevertheless comprise only environmental sequences. Our results demonstrate that other types of reference partitions are also of use. For apparently highly specialized symbiont (mutualist or parasitic) organisms, agreement with the host taxonomy is a good candidate. In the case of our downy mildew ITS dataset, using the GenBank host entries, if pre-processed in a manner similar to the taxonomy, resulted in exactly the same optimal parameters than the taxonomy-based optimization. If several suitable reference partitions are present (e.g. a matrix of morphological characters or alternative codings of the same underlying data to represent uncertainty), the MRI can be averaged between the distinct partitions, as already implemented in the optsil program.

### Outlook for the molecular taxonomy of the downy mildews

While phylogenetic reconstruction is necessary to identify monophyletic units, it only provides criteria for grouping, not for ranking. It was a common observation in molecular phylogenetic studies on DM that near-terminal clades (subtrees) within the trees were comprised of collections from identical or closely related host plants [40]–[44], [67], [68], [70], [72]. In the most comprehensive molecular phylogenetic study on a DM genus up to now, Göker et al. [44] have shown in detail how a combination of molecular and host characters can be applied to obtain a stable DM taxonomy, even though it is mostly impossible to separate species morphologically. However, because the terminal subtrees containing DM samples with uniform host specificity had to be selected by manual inspection of the trees, an element of arbitrariness remained in the species concept put forward in our earlier studies [40], [43], [44]. Clustering optimization using the hosts as a reference partitions provides an algorithmic solution to this problem, as it allows one to obtain taxonomic units (as estimates for DM species) in a fully automated way, given the names of the plant hosts (which are much easier to determine morphologically than the *Peronospora* species) and a distance matrix. As demonstrated in the present study, the resulting clustering parameters are identical to those obtained by using the current taxonomy as the reference, and the resulting clusters appear as monophyletic in phylogenetic trees (Figs. 3–5). Thus, clustering optimization can be used to define DM species as monophyletic units that are characterized by a specific set of (almost always) closely related host plants and by a comparable genetic diversity, thus deserving the same taxonomic rank.

As shown here, clustering optimization is not less conservative than traditional taxonomy; rather, some *Peronospora* taxa were split (Ia) while others were fused (IIa). Such discrepancies can usually be attributed to insufficient information on host specificity [43], [44]. In fact, the vast majority of the sequences subjected to clustering optimization either does not show a discrepancy between traditional and molecular taxonomy or a discrepancy that can be interpreted biologically (Ia or IIa). Splitting implies that the old species name has to be assigned to one of the splitting products, a decision that must rely on the type host of the species. Thus, a major obstacle for an updated *Peronospora* nomenclature that integrates the recent molecular taxonomic results is the lack of type hosts for all the species described in Gäumann's monograph [35] which are based on collections from more than one host species, as he did not designate types. Therefore, all these (sometimes numerous) collections cited in the protologue are authentic collections, which do not represent types unless a lectotype is chosen amongst them. Lectotypification is urgently needed for these taxa to provide nomenclatural stability, but will require thorough studies. Accordingly, to establish an enhanced taxonomic system for the DM species, research should now focus on the observed discrepancies between the current nomenclature and the molecular taxonomic units obtained using the optimized settings.

### Conclusion

Beyond its obvious suitability for molecular taxonomy in its current version, the suggested clustering optimization algorithm is an excellent starting point for further methodological improvements. For instance, clustering algorithms other than linkage clustering can be tested. Erroneous sequence data present a problem for molecular identification that is based on fixed threshold values, and a number of falsely separated clusters caused by sequencing errors were observed in the present dataset (supporting file S2). Similar flaws may occur in the case of considerable differences in genetic divergence between the species, particularly if high *F* values are optimal. However, these problems are neither specific to downy mildew molecular taxonomy, nor to the optimization principle, but are also present in the many studies that apply arbitrary thresholds. In addition to further clustering algorithms, alternative measures for the agreement between clustering results and reference data are of interest. However, most likely all of these improvements will be based on the same principle, i.e. to optimize the agreement between molecular classification and external information. Since our optimization approach shows so much promise for downy mildew taxonomy, we expect it to be of general use. Whether its strict objectivity and reproducibility will help to dispel some of the criticism on the “unholy” aspects of molecular taxonomy [19], remains to be seen. At the very least, it is likely that the adaptation of molecular taxonomy to biologically informative reference data, without relying on the assumptions that the latter are 100% reasonable, is a concept that is appealing for both traditional and molecular taxonomists.

## Supporting Information

File S1 Includes the inferred alignments in FASTA, the trees in Newick and selected distance matrices in extended PHYLIP format.
(0.60 MB ZIP)

File S2 Contains the GenBank accession numbers of the sequences under study and all other information extracted from the GenBank files (including the reference partitions), the taxonomy of the host plants, the complete clustering optimization outcome, its taxonomic interpretation and corresponding literature references.
(0.25 MB XLS)

File S3 Shows the results regarding the effect of alternative alignment programs and distance formulae.
(0.14 MB PDF)

File S4 Contains the illustration of the taxon jackknifing and reference partition random permutation experiments.
(0.15 MB PDF)

## References

[1] Blaxter M, Floyd R (2003). Molecular taxonomics for biodiversity surveys: already a reality.. Trends Ecol Evol.

[2] Blaxter M, Mann J, Chapman T, Thomas F, Whitton C (2005). Defining operational taxonomic units using DNA barcode data.. Philos Trans R Soc London, Ser B.

[3] Daniell TJ, Husband R, Fitter AH, Young JPW (2001). Molecular diversity of arbuscular mycorrhizal fungi colonising arable crops.. FEMS Microbiol Ecol.

[4] Floyd R, Abebe E, Papert A, Blaxter M (2002). Molecular barcodes for soil nematode identification.. Mol Ecol.

[5] Helgason T, Watson IJ, Young JPW (2003). Phylogeny of the Glomerales and Diversisporales (Fungi: Glomeromycota) from actin and elongation factor 1-alpha sequences.. FEMS Microbiol Ecol.

[6] Husband R, Herre EA, Turner SL, Gallery R, Young JPW (2002). Molecular diversity of arbuscular mycorrhizal fungi and pattern of host association over time and space in a tropical forest.. Mol Ecol.

[7] Schloss PD, Handelsman J (2004). Status of the microbial census.. Microbiology and Molecular Biology Reviews.

[8] Schloss PD, Handelsman J (2005). Introducing DOTUR, a computer program for defining operational taxonomic units and estimating species richness.. Appl Environ Microbiol.

[9] Vandenkoornhuyse P, Husband R, Daniell TJ, Watson IJ, Duck JM (2002). Arbuscular mycorrhizal community composition associated with two plant species in a grassland ecosystem.. Mol Ecol.

[10] Wubet T, Weiß M, Kottke I, Teketay D, Oberwinkler F (2006). Phylogenetic analysis of nuclear small subunit rDNA sequences suggests that the endangered African Pencil Cedar, *Juniperus procera*, is associated with distinct members of Glomeraceae.. Mycol Res.

[11] Knowlton N (1993). Sibling species in the sea.. Annu Rev Ecol Syst.

[12] Tringe SG, Rubin EM (2005). Metagenomics: DNA sequencing of environmental samples.. Nat Rev Genet.

[13] Rusch DB, Halpern AL, Sutton G, Heidelberg KB, Williamson S (2007). The Sorcerer II Global Ocean Sampling expedition: northwest Atlantic through eastern tropical Pacific.. PLoS Biol.

[14] Belbahri L, Calmin G, Pawlowski J, Lefort F (2005). Phylogenetic analysis and real time PCR detection of a presumably undescribed *Peronospora* species on sweet basil and sage.. Mycol Res.

[15] Landa BB, Montes-Borrego M, Muñoz-Ledesma FJ, Jiménez-Díaz RM (2007). Phylogenetic analysis of downy mildew pathogens of opium poppy and PCR-based in planta and seed detection of *Peronospora arborescens*.. Phytopathology.

[16] Hebert P, Gregory T (2005). The promise of DNA barcoding for taxonomy.. Syst Biol.

[17] Kress WJ, Erickson DL (2008). DNA Barcoding - a Windfall for Tropical Biology?. Biotropica.

[18] Will K, Mishler B, Wheeler Q (2005). The perils of DNA barcoding and the need for integrative taxonomy.. Syst Biol.

[19] DeSalle R, Egan MG, Siddall M (2005). The unholy trinity: Taxonomy, species delimitation and DNA barcoding.. Philos Trans R Soc London Ser B.

[20] Ferguson JWH (2002). On the use of genetic divergence for identifying species.. Biol J Linn Soc.

[21] Will KW, Rubinoff D (2004). Myth of the molecule: DNA barcodes for species cannot replace morphology for identification and classification.. Cladistics.

[22] Schloss P, Handelsman J (2006). Toward a census of bacteria in soil.. PLoS Computational Biology.

[23] Sokal RR, Sneath PHA (1963). Principles of Numerical Taxonomy..

[24] Legendre P, Legendre L (1998). Numerical ecology, 2nd English edition..

[25] Nilsson RH, Kristiansson E, Ryberg M, Hallenberg N, Larsson K-H (2008). Intraspecific ITS Variability in the Kingdom Fungi as Expressed in the International Sequence Databases and Its Implications for Molecular Species Identification.. Evolutionary Bioinformatics.

[26] Abdo Z, Golding B (2007). A step toward barcoding life: A model-based, decision-theoretic method to assign genes to preexisting species groups.. Syst Biol.

[27] Nielsen R, Matz M (2006). Statistical approaches for DNA barcoding.. Syst Biol.

[28] Zhang AB, Sikes DS, Muster C, Li SQ (2008). Inferring species membership using DNA sequences with back-propagation neural networks.. Syst Biol.

[29] Linnen CR, Farrell BD (2008). Comparison of methods for species-tree inference in the sawfly genus *Neodiprion* (Hymenoptera: Diprionidae).. Syst Biol.

[30] Felsenstein J (2004). Inferring Phylogenies..

[31] Dick MW (2001). Straminipilous Fungi, Systematics of the Peronosporomycetes, including accounts of the marine Straminipilous protists, the Plasmodiophorids and similar organisms..

[32] Hall GS (1996). Modern approaches to species concepts in downy mildews.. Plant Pathology.

[33] de Bary A (1863). Recherches sur le developpement de quelques champignons parasites.. Annales des Sciences Naturelles, Botanique, sér 4.

[34] Gäumann E (1918). Über die Formen der *Peronospora parasitica* (Pers.) Fries.. Beihefte zum Botanischen Centralblatt.

[35] Gäumann E (1923). Beiträge zu einer Monographie der Gattung *Peronospora* Corda.. Beiträge zur Kryptogamenflora der Schweiz.

[36] Gustavsson A (1959). Studies on Nordic Peronosporas. I. Taxonomic revision.. Opera Botanica.

[37] Gustavsson A (1959). Studies on Nordic Peronosporas. II. General account.. Opera Botanica.

[38] Săvulescu T (1948). Les espèces de *Peronospora* Corda de la Roumainie.. Sydowia.

[39] Yerkes WD, Shaw CG (1959). Taxonomy of *Peronospora* species on Cruciferae and Chenopodiaceae.. Phytopathology.

[40] García-Blázquez G, Göker M, Voglmayr H, Martín MP, Tellería MT (2008). Phylogeny of *Peronospora*, parasitic of Fabaceae, based on ITS sequences.. Mycol Res.

[41] Voglmayr H (2003). Phylogenetic relationships of *Peronospora* and related genera based on nuclear ribosomal ITS sequences.. Mycol Res.

[42] Choi Y-J, Hong S-B, Shin H-D (2003). Diversity of the *Hyaloperonospora parasitica* complex from core brassicaceous hosts based on ITS rDNA sequences.. Mycol Res.

[43] Göker M, Riethmüller A, Voglmayr H, Weiß M, Oberwinkler F (2004). Phylogeny of *Hyaloperonospora* based on nuclear ribosomal internal transcribed spacer sequences.. Mycological Progress.

[44] Göker M, Voglmayr H, García-Blázquez G, Oberwinkler F (2009). Species delimitation in downy mildews: the case of *Hyaloperonospora* in the light of nuclear ribosomal ITS and LSU sequences.. Mycol Res.

[45] Voglmayr H, Constantinescu O (2007). Revision and reclassification of three *Plasmopara* species based on morphological and molecular phylogenetic data.. Mycol Res.

[46] Voglmayr H, Fatehi J, Constantinescu O (2006). Revision of *Plasmopara* (Chromista, Peronosporales) parasitic on Geraniaceae.. Mycol Res.

[47] Choi Y-J, Hong S-B, Shin H-D (2005). A re-consideration of *Pseudoperonospora cubensis* and *P. humuli* based on molecular and morphological data.. Mycol Res.

[48] Göker M, Voglmayr H, Riethmüller A, Weiß M, Oberwinkler F (2003). Taxonomic aspects of Peronosporaceae inferred from Bayesian molecular phylogenetics.. Can J Bot.

[49] Göker M, Voglmayr H, Riethmüller A, Oberwinkler F (2007). How do obligate parasites evolve? A multi-gene phylogenetic analysis of downy mildews.. Fungal Genet Biol.

[50] White TJ, Bruns T, Lee S, Taylor J, Innis MA, Gelfand H, Sninsky JS, White TJ (1990). Amplification and direct sequencing of fungal ribosomal RNA genes for phylogenetics.. PCR Protocols: a guide to methods and applications.

[51] Martín MP, Winka K (2000). Alternative methods of extracting and amplifying DNA from lichens.. Lichenologist.

[52] Lee C, Grasso C, Sharlow M (2002). Multiple sequence alignment using partial order graphs.. Bioinformatics.

[53] Swofford DL (2002). PAUP*: Phylogenetic Analysis Using Parsimony (*and Other Methods), Version 4.0 b10..

[54] Stamatakis A (2006). RAxML-VI-HPC: Maximum-Likelihood-based phylogenetic analyses with thousands of taxa and mixed models.. Bioinformatics.

[55] Estabrook GF (1966). A mathematical model in graph theory for biological classification.. J Theor Biol.

[56] Meier R, Shiyang K, Vaidya G, Ng PKL (2006). DNA barcoding and taxonomy in Diptera: A tale of high intraspecific variability and low identification success.. Syst Biol.

[57] Rand WM (1971). Objective criteria for the evaluation of clustering methods.. Journal of the American Statistical Association.

[58] Hubert L, Arabie P (1985). Comparing partitions.. J Classif.

[59] Lanyon S (1985). Detecting internal inconsistencies in distance data.. Syst Zool.

[60] Farris JS, Platnick N, Funk V (1983). The logical basis of phylogenetic analysis.. Advances in Cladistics, Volume 2.

[61] Sokal RR, Michener CD A statistical method for evaluating systematic relationships.. Univ Kansas Sci Bull 1958.

[62] Stamatakis A, Hoover P, Rougemont J (2008). A rapid bootstrap algorithm for the RAxML web-servers.. Syst Biol.

[63] Stamatakis A (2006). Phylogenetic models of rate heterogeneity: a high performance computing perspective.. Proceedings 20th IEEE International Parallel & Distributed Processing Symposium..

[64] Lee MSY (2001). Unalignable sequences and molecular evolution.. Trends Ecol Evol.

[65] Kemler M, Göker M, Oberwinkler F, Begerow D (2006). Implications of molecular characters for the phylogeny of the Microbotryaceae (Basidiomycota: Urediniomycetes).. BMC Evol Biol.

[66] Fitch WM (1971). Towards defining the course of evolution: minimal change for a specified tree topology.. Syst Zool.

[67] Choi Y-J, Hong S-B, Shin H-D (2007). Re-consideration of *Peronospora farinosa* infecting *Spinacia oleracea* as distinct species, *Peronospora effusa*.. Mycol Res.

[68] Choi Y-J, Denchev CM, Shin H-D (2008). Morphological and molecular analysis support the existence of host-specific *Peronospora* species infecting *Chenopodium*.. Mycopathologia.

[69] Endo Y, Choi B-H, Ohashi H, Delgado-Salinas A (2008). Phylogenetic relationships of new world *Vicia* (Leguminosae) inferred from nrDNA internal transcribed spacer sequences and floral characters.. Systematic Botany.

[70] Cunnington JH (2006). DNA sequence variation supports multiple host-specialised taxa in the *Peronospora viciae* complex (Chromista: Peronosporales).. Nova Hedwigia.

[71] Fior S, Karis PO, Casazza G, Minuto L, Sala F (2006). Molecular phylogeny of the Caryophyllaceae (Caryophyllales) inferred from chloroplast matk and nuclear rDNA ITS sequences.. Amer J Bot.

[72] Voglmayr H, Riethmüller A, Göker M, Weiß M, Oberwinkler F (2004). Phylogenetic relationships of Plasmopara, Bremia and other genera of downy mildews with pyriform haustoria based on Bayesian analysis of partial LSU rDNA sequence data.. Mycol Res.

