# Supplementary material for: Molecular Taxonomy of Phytopathogenic Fungi: A Case Study in Peronospora
Source: PLoS One. 2009 Jul 29;4(7):e6319. doi: 10.1371/journal.pone.0006319 (PMC2712678; doi:10.1371/journal.pone.0006319)
Supplement: File S3 — Shows the results regarding the effect of alternative alignment programs and distance formulae. (0.14 MB PDF) [file pone.0006319.s003.pdf]

Supplementary material to Göker, García-Blázquez, Voglmayr, Tellería & Martín, „*Molecular taxonomy of phytopathogenic fungi: a case study in* Peronospora“: Effect of distinct sequence alignments and distance formulae

Introduction

The computation of the distance matrices may cause considerable methodological problems because of alignment ambiguity (Lake 1991, Morrison & Ellis 1997), particularly in the case of highly divergent markers, and rate heterogeneity between sites. While more complex distance formulae than the uncorrected („p“) distances mainly used in molecular taxonomy may accommodate for rate heterogeneity (e.g., Swofford et al. 1996), multiple analysis, i.e. the application of distinct alignment programs or parameters (Lee 2001, Kemler et al. 2006), can be used to cope with alignment ambiguity. It thus should be possible to extend the clustering optimization principle to select the best distance formula and multiple sequence alignment approach.

Methods

To assess the effect of DNA alignment on the clustering (and phylogenetic inference) results, additional multiple sequence alignments were inferred with four other software packages, CLUSTALW version 1.81 (Thompson et al. 1997), KALIGN version 2.03 (Lassmann & Sonnhammer 2005), MAFFT version 6.24 (Katoh et al. 2005), MUSCLE (Edgar 2004), as well as with POA in global scoring mode (using the command-line switch -do_global; henceforth referred to as POAGLO), and additional distance matrices inferred from these alignments. To assess the impact of other distance formulae, PAUP* was used to calculate Jukes-Cantor; Felsenstein 1981; Kimura-2-parameter; Felsenstein 1984; Kimura-3-parameter; Tamura-Nei; General Time-Reversible; and LogDet distances, too (see Swofford et al. 1996 for a survey of these distance methods). As far as possible (i.e., except for P and LogDet distances), we combined the formulae not only with equal, but also with gamma-distributed substitution rates, using an alpha parameter of 0.5 (Swofford et al. 1996). The according PAUP* command was DSET DIST = {P / JC / F81 / K2P / F84 / K3P / TAMNEI / GTR / LOGDET} MISSDIST = IGNORE RATES = {EQUAL / GAMMA} SHAPE = 0.5; all other settings corresponded to the default values. Furthermore, distances were calculated under the maximum likelihood (ML) criterion with RAxML version 7.04 (Stamatakis 2006, Stamatakis et al. 2008) in conjunction with the GTRMIX model approximation (command-line switches -m GTRMIX -f x). Accordingly, 108 alignment-based distance approaches were subjected to clustering optimization in the same way than the GBDP formulae, and it was reported whether other alignment approaches and/or distance formulae would result in a significantly better result than the main analysis based on the fast POA alignment and simple uncorrected distances.

Results

Using other alignment programs (features of the inferred multiple sequence alignments are listed below) and/or distance formulae did not result in considerably higher MRI values; rather, improvements were restricted to the third position after the decimal point. Best formula for the POA alignment was GTR+GAMMA (F = 1.0, T = 0.00770, MRI = 0.85721) in the case of taxonomy-based and F81+GAMMA (F = 1.0, T = 0.00760, MRI = 0.85868) in the case of the host-based optimization. The globally best combination of alignment and distance formula was MAFFT+F81 (F = 0.25, T = 0.00430, MRI = 0.85724) in the case of taxonomy-based and POA+F81+GAMMA (as

above) in the case of the host-based optimization. The best MRI values obtained for the POA alignment and all distance formulae, dependent on the tested F values, is shown in Figs. 1 and 2, corresponding to either reference partition. While an additional local maximum is present in the case of taxonomy-based optimization for F = 0.25 and F = 0.30, F = 1.0 gives far superior MRI values than any other F value for both partitions.

The best tree inferred with RAxML from the POA alignment had a log Likelihood of -16392.00; other software resulted in longer or shorter alignments and distinct log Likelihood values (CLUSTALW: 1,665/-19474.69; KALIGN: 1,975/-19177.46; MAFFT: 2,073/18562.37; MUSCLE: 2,384/-20477.71; POAGLO: 2,128/-17003.01 bp). Length differences were mainly caused by the treatment of the ITS1 insertions of the *Trifolium* parasites (García-Blázquez et al. 2008); total length was partly due to long SSU fragments in some accessions.

Additional references (not covered by the main manuscript)

Edgar, R.C. 2004 MUSCLE: multiple sequence alignment with high accuracy and high throughput. *Nucleic Acids Res.* 32, 1792-1797.

Katoh, K., Kuma, K., Toh, H. & Miyata, T. 2005 MAFFT version 5: improvement in accuracy of multiple sequence alignment. *Nucleic Acids Res.* 33, 511-518.

Lake, J.A. 1991 The order of sequence alignment can bias the selection of tree topology. *Mol. Biol. Evol.* 8, 378-385.

Lassmann, T. & Sonnhammer, E.L.L. 2005 Kalign - an accurate and fast multiple sequence alignment algorithm. *BMC Bioinformatics* 6, 298.

Morrison, D.A. & Ellis, J.T. 1997 Effects of nucleotide sequence alignment on phylogeny estimation: a case study of 18S rDNAs of Apicomplexa. *Mol. Biol. Evol.* 14, 428-441.

Swofford, D. L., Olsen, G. J., Waddell, P. J. & Hillis, D. M. 1996 Phylogenetic inference. In: *Molecular systematics* (D. M. Hillis, C. Moritz & B. K. Mable, eds): 407–514. Sinauer Associates, Sunderland, MA.

Thompson, J.D., Gibson, J.T., Plewniak, F., Jeanmougin, F. & Higgins, D.G. 1997 The ClustalX windows interface: flexible strategies for multiple sequence alignment aided by quality analysis tools. *Nucleic Acids Res.* 24, 4876-4882.

Figures

Figure 1 (next page). Best MRI values (y axis) obtained for the POA alignment and all distance formulae, dependent on the tested F values (x axis), if the *Peronospora*/*Pseudoperonospora* taxonomy is used as reference partition.

Figure 2 (page after next page). Best MRI values (y axis) obtained for the POA alignment and all distance formulae, dependent on the tested F values (x axis), if the taxonomy of the plant hosts is used as reference partition.

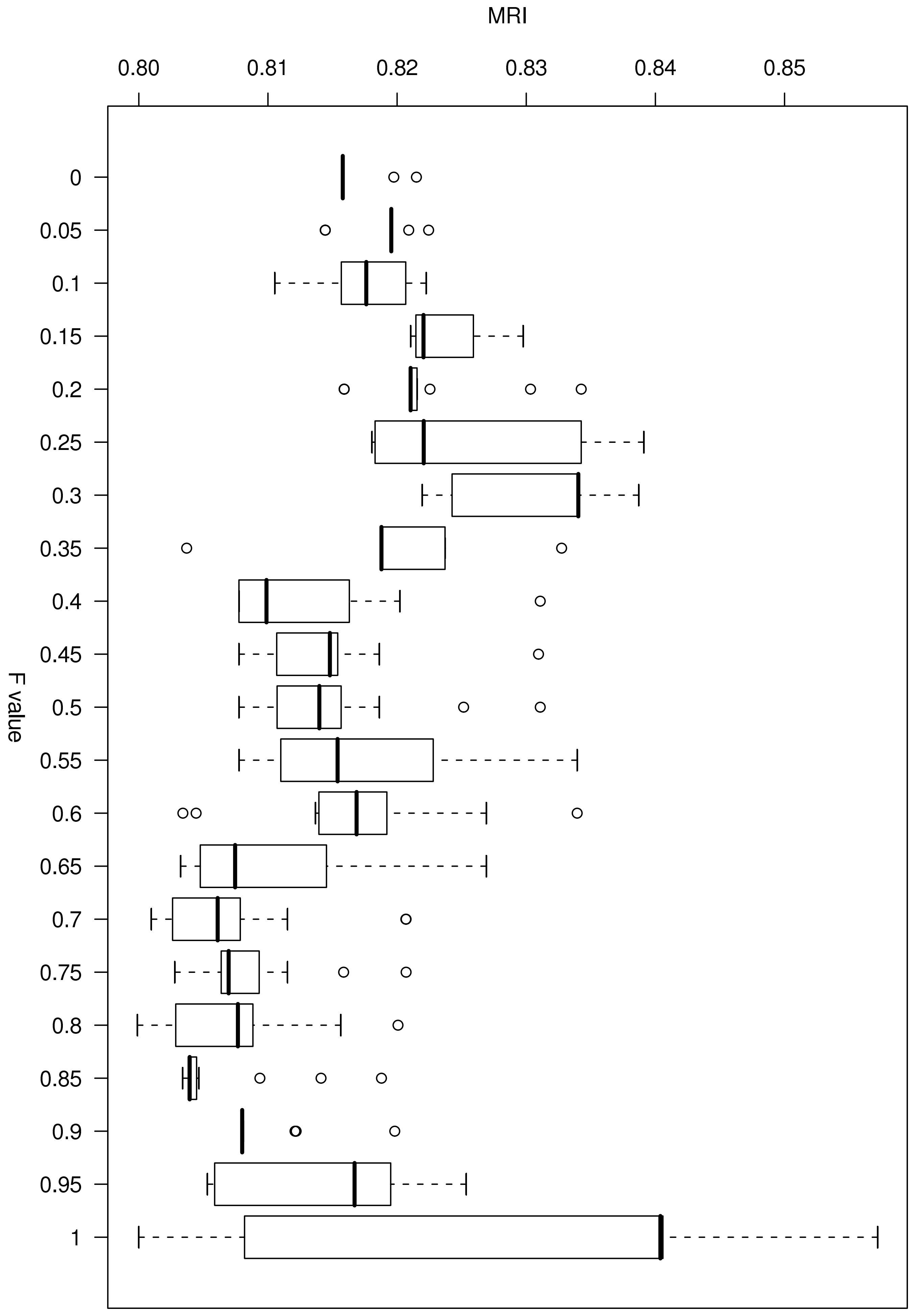
MRI
0.80
0.81
0.82
0.83
0.84
0.85
F value
0
0.05
0.1
0.15
0.2
0.25
0.3
0.35
0.4
0.45
0.5
0.55
0.6
0.65
0.7
0.75
0.8
0.85
0.9
0.95
1

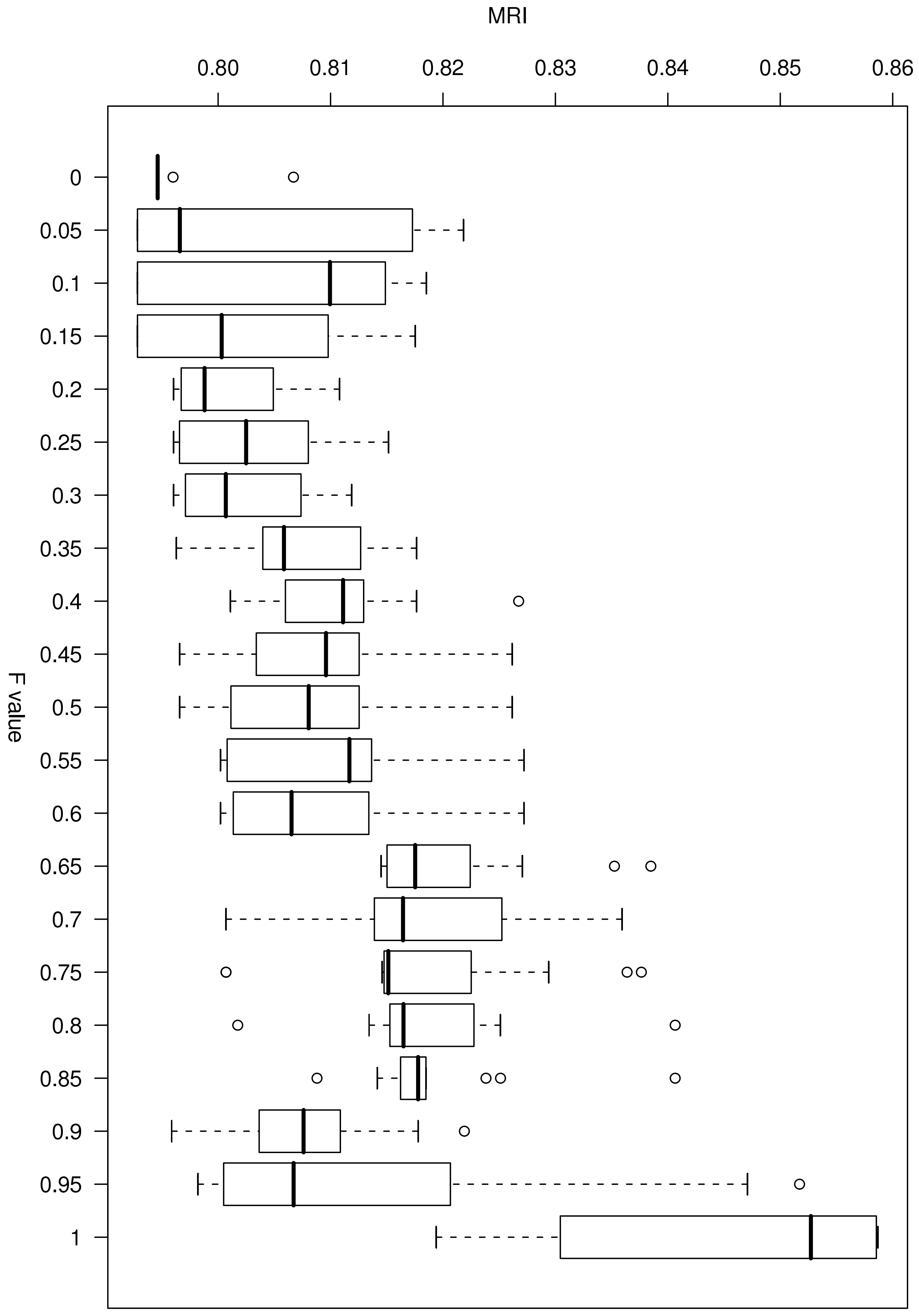
MRI
0.80
0.81
0.82
0.83
0.84
0.85
0.86
F value
0
0.05
0.1
0.15
0.2
0.25
0.3
0.35
0.4
0.45
0.5
0.55
0.6
0.65
0.7
0.75
0.8
0.85
0.9
0.95
1
