# Supplementary material for: Molecular Taxonomy of Phytopathogenic Fungi: A Case Study in Peronospora
Source: PLoS One. 2009 Jul 29;4(7):e6319. doi: 10.1371/journal.pone.0006319 (PMC2712678; doi:10.1371/journal.pone.0006319)
Supplement: File S4 — Contains the illustration of the taxon jackknifing and reference partition random permutation experiments. (0.15 MB PDF) [file pone.0006319.s004.pdf]

Supplementary Material to Göker, Garcia-Blazquez, Voglmayr, Telleria & Martin, "*Molecular taxonomy of phytopathogenic fungi - a case study in* Peronospora": Robustness of parameter identification in clustering optimization

Figures 1-5: Experiments with taxon jackknifing to test for robustness regarding taxon sampling if the taxonomy-based reference partition is used. A defined proportion of the sequences was removed before optimizing the parameters. Sequences to be removed were selected at random in each of the 1,000 taxon jackknifing replicates.

Figure 1: Dependency of the smallest optimal F values on the proportion of sequences randomly deleted before optimization.

Figure 2: Dependency of the largest optimal F values on the proportion of sequences randomly deleted before optimization.

Figure 3: Dependency of the smallest optimal threshold values on the proportion of sequences randomly deleted before optimization.

Figure 4: Dependency of the largest optimal threshold values on the proportion of sequences randomly deleted before optimization.

Figure 5: Dependency of obtained MRI values on the proportion of sequences randomly deleted before optimization.

Figures 6-10: Experiments with random permutations to test for robustness regarding the taxonomy-based reference partition. A defined proportion of errors was introduced in the reference partition before optimizing the parameters. Errors to be introduced were selected at random in each of the 1,000 replicates.

Figure 6: Dependency of the smallest optimal F values on the proportion of errors randomly introduced before optimization.

Figure 7: Dependency of the largest optimal F values on the proportion of errors randomly introduced before optimization.

Figure 8: Dependency of the smallest optimal threshold values on the proportion of errors randomly introduced before optimization.

Figure 9: Dependency of the largest optimal threshold values on the proportion of errors randomly introduced before optimization.

Figure 10: Dependency of obtained MRI values on the proportion of errors randomly introduced before optimization.

Figures 11-15: Experiments with taxon jackknifing to test for robustness regarding taxon sampling if the host-based reference partition is used. A defined proportion of the sequences was removed before optimizing the parameters. Sequences to be removed were selected at random in each of the 1,000 taxon jackknifing replicates.

Figure 11: Dependency of the smallest optimal F values on the proportion of sequences randomly deleted before optimization.

Figure 12: Dependency of the largest optimal F values on the proportion of sequences randomly deleted before optimization.

Figure 13: Dependency of the smallest optimal threshold values on the proportion of sequences randomly deleted before optimization.

Figure 14: Dependency of the largest optimal threshold values on the proportion of sequences randomly deleted before optimization.

Figure 15: Dependency of obtained MRI values on the proportion of sequences randomly deleted before optimization.

Figures 16-20: Experiments with random permutations to test for robustness regarding the taxonomy-based reference partition. A defined proportion of errors was introduced in the reference partition before optimizing the parameters. Errors to be introduced were selected at random in each of the 1,000 replicates.

Figure 16: Dependency of the smallest optimal F values on the proportion of errors randomly introduced before optimization.

Figure 17: Dependency of the largest optimal F values on the proportion of errors randomly introduced before optimization.

Figure 18: Dependency of the smallest optimal threshold values on the proportion of errors randomly introduced before optimization.

Figure 19: Dependency of the largest optimal threshold values on the proportion of errors randomly introduced before optimization.

Figure 20: Dependency of obtained MRI values on the proportion of errors randomly introduced before optimization.

Figure 1

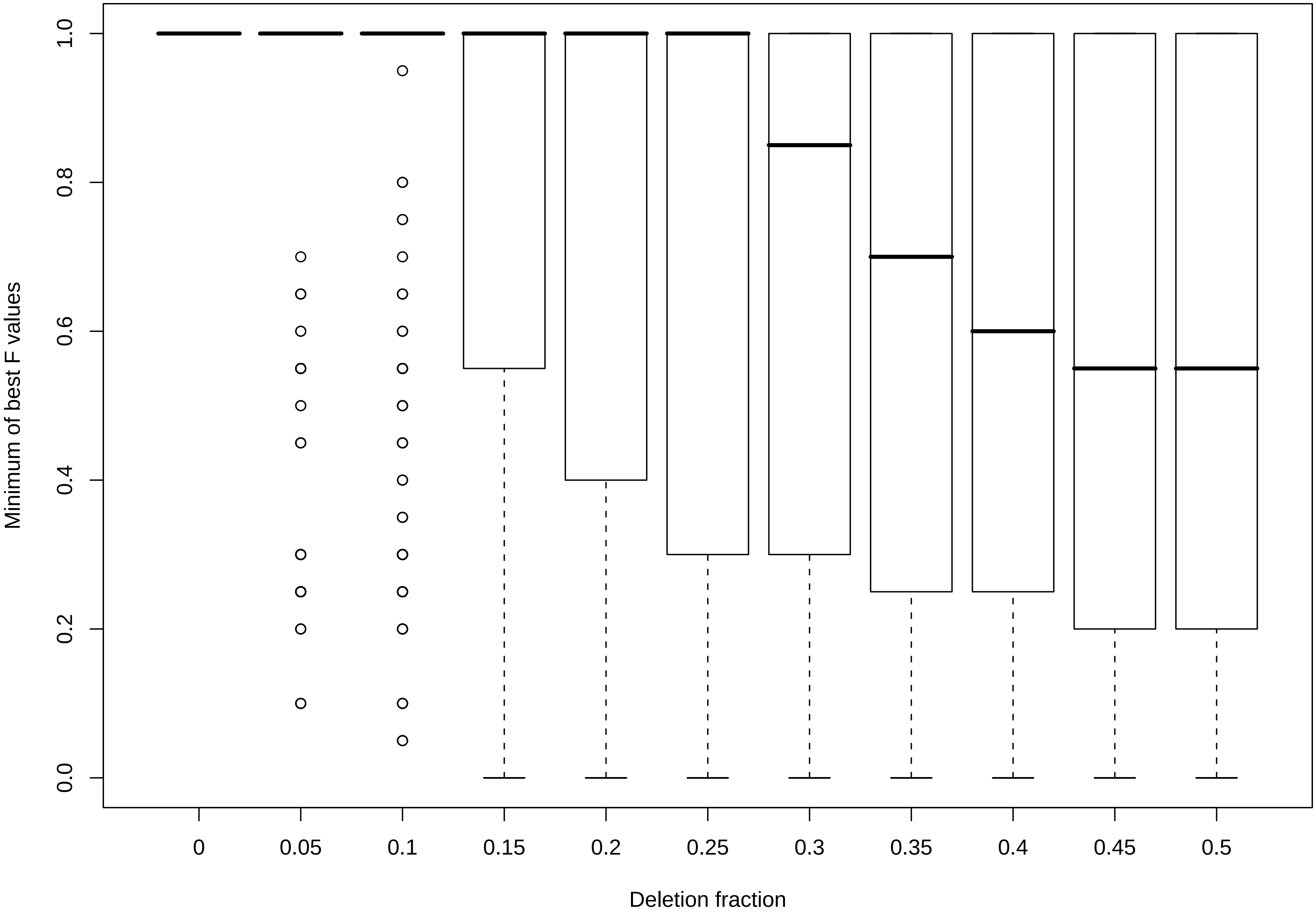
Minimum of best F values
0.0
0.2
0.4
0.6
0.8
1.0
0
0.05
0.1
0.15
0.2
0.25
0.3
0.35
0.4
0.45
0.5
Deletion fraction

Figure 2

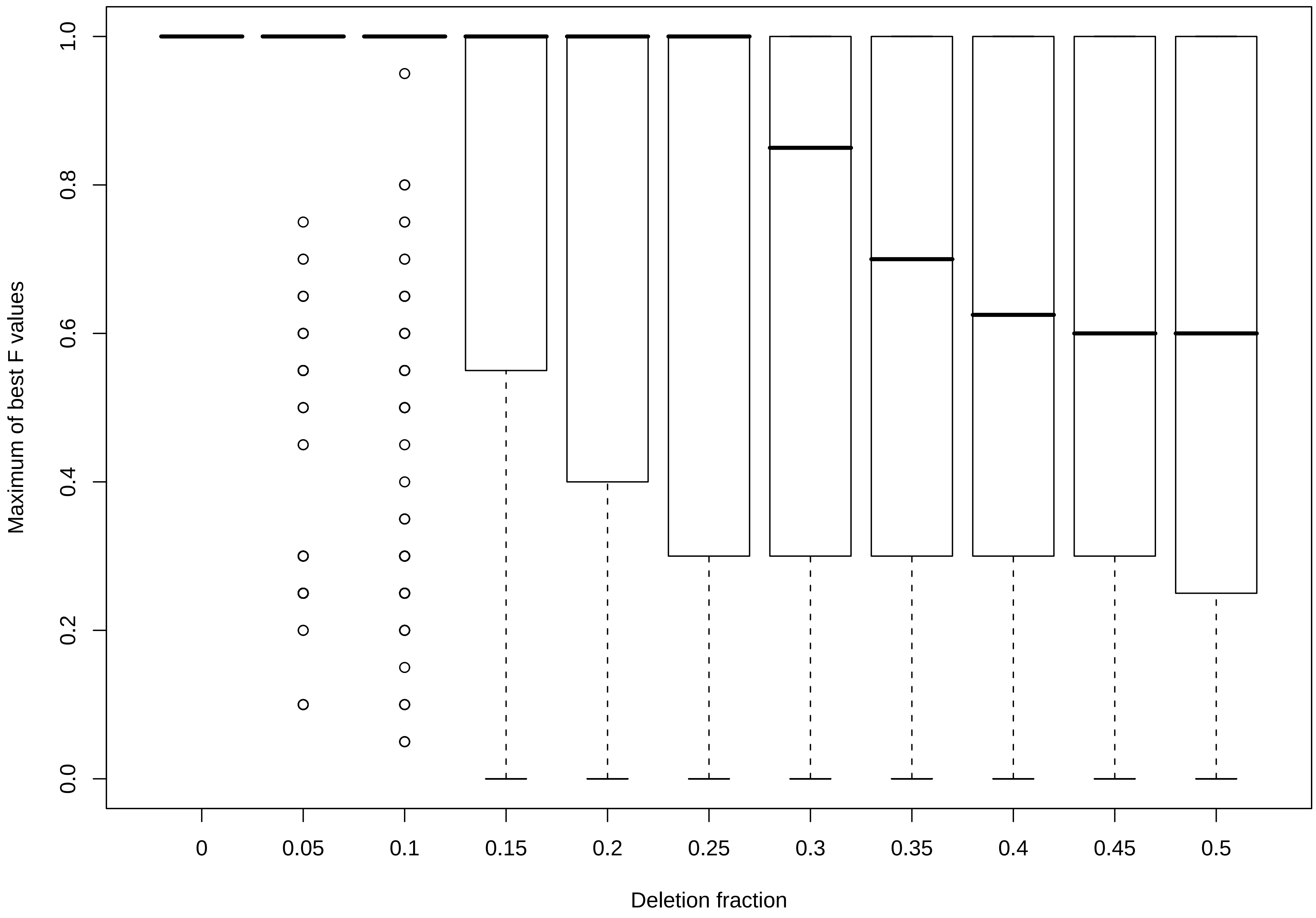

1.0
0.8
0.6
0.4
0.2
0.0
Maximum of best F values
0
0.05
0.1
0.15
0.2
0.25
0.3
0.35
0.4
0.45
0.5
Deletion fraction

Figure 3

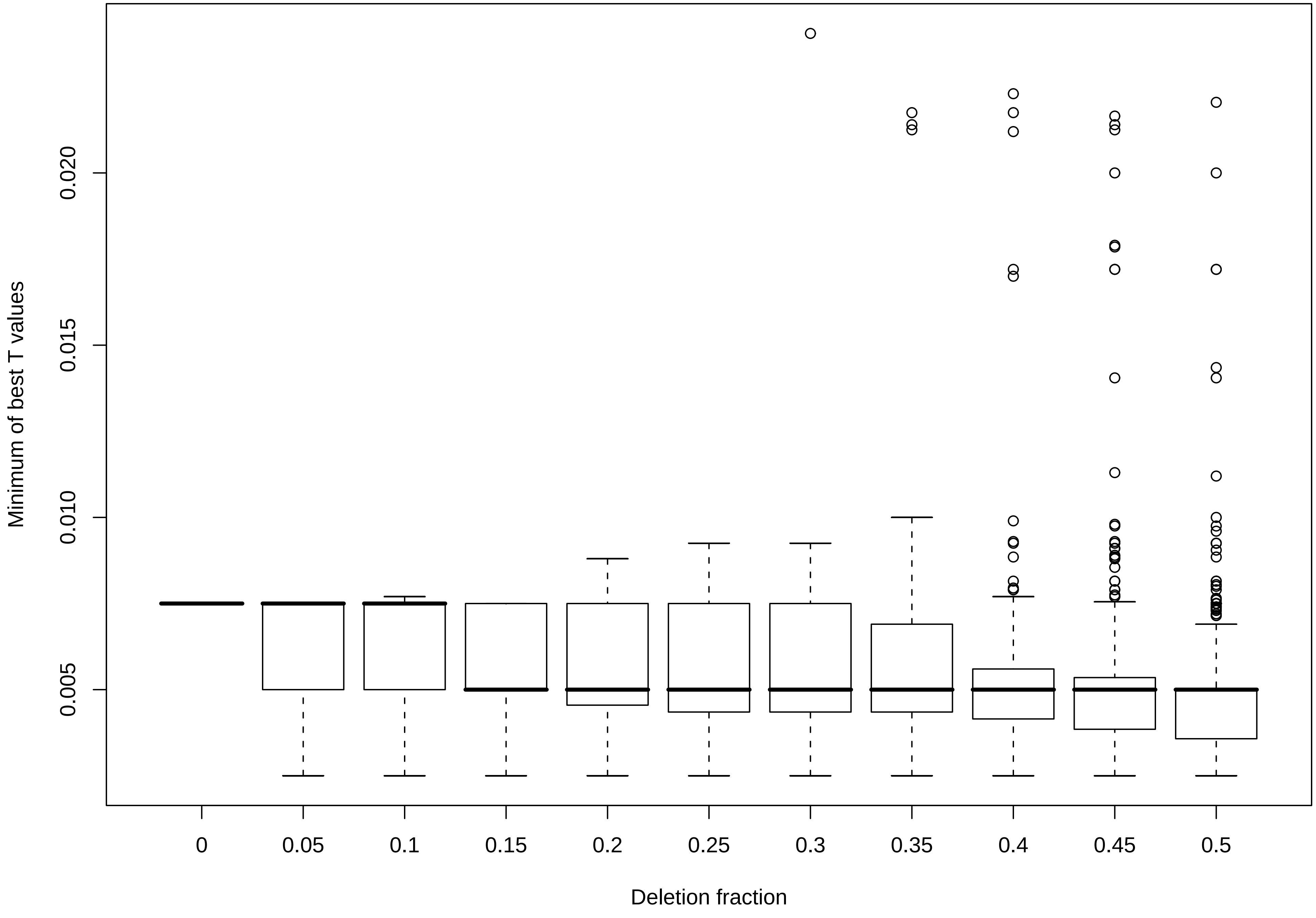
Minimum of best T values
0.005
0.010
0.015
0.020
0
0.05
0.1
0.15
0.2
0.25
0.3
0.35
0.4
0.45
0.5
Deletion fraction

Figure 4

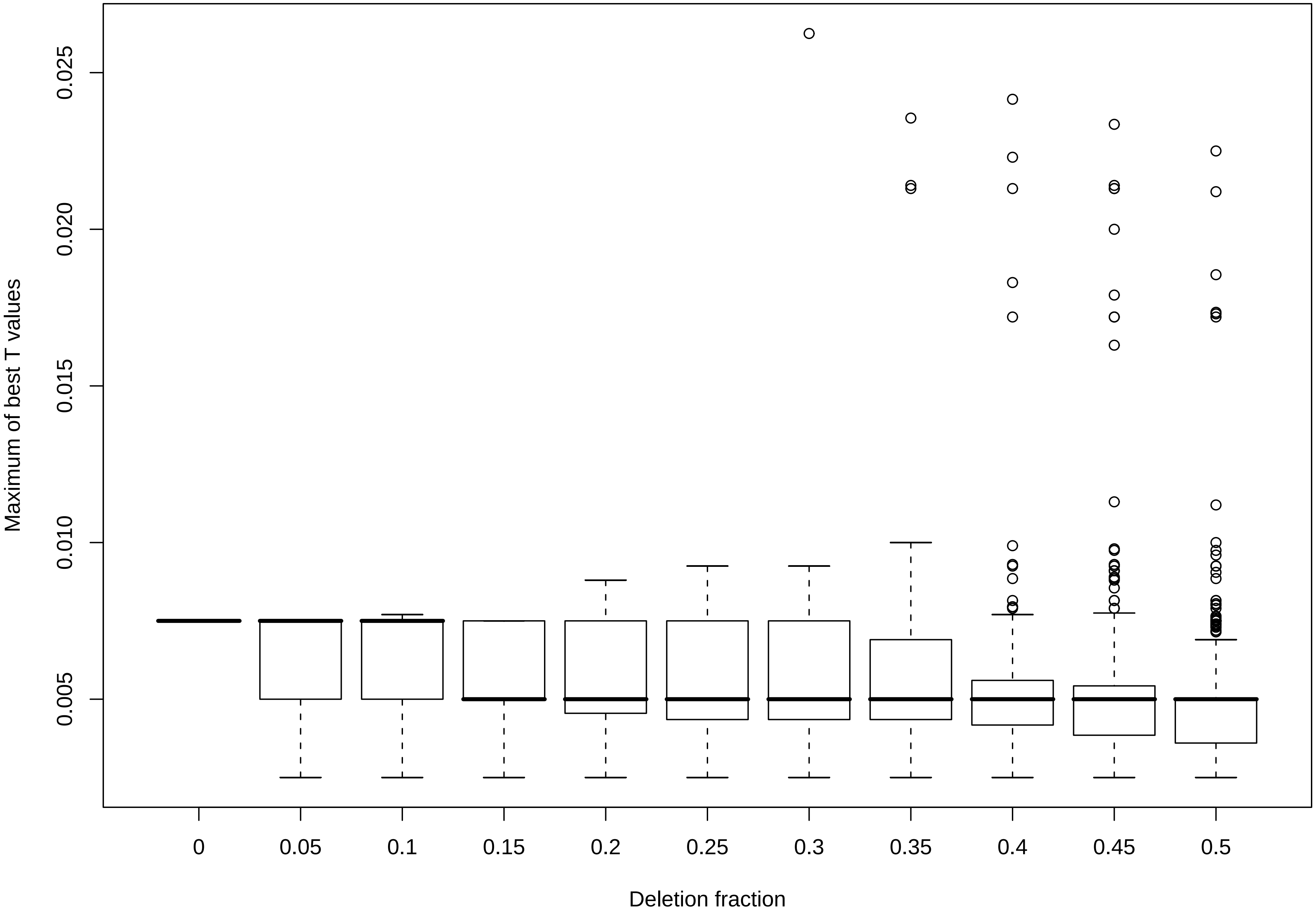

Maximum of best T values
0.005
0.010
0.015
0.020
0.025
0
0.05
0.1
0.15
0.2
0.25
0.3
0.35
0.4
0.45
0.5
Deletion fraction

Figure 5

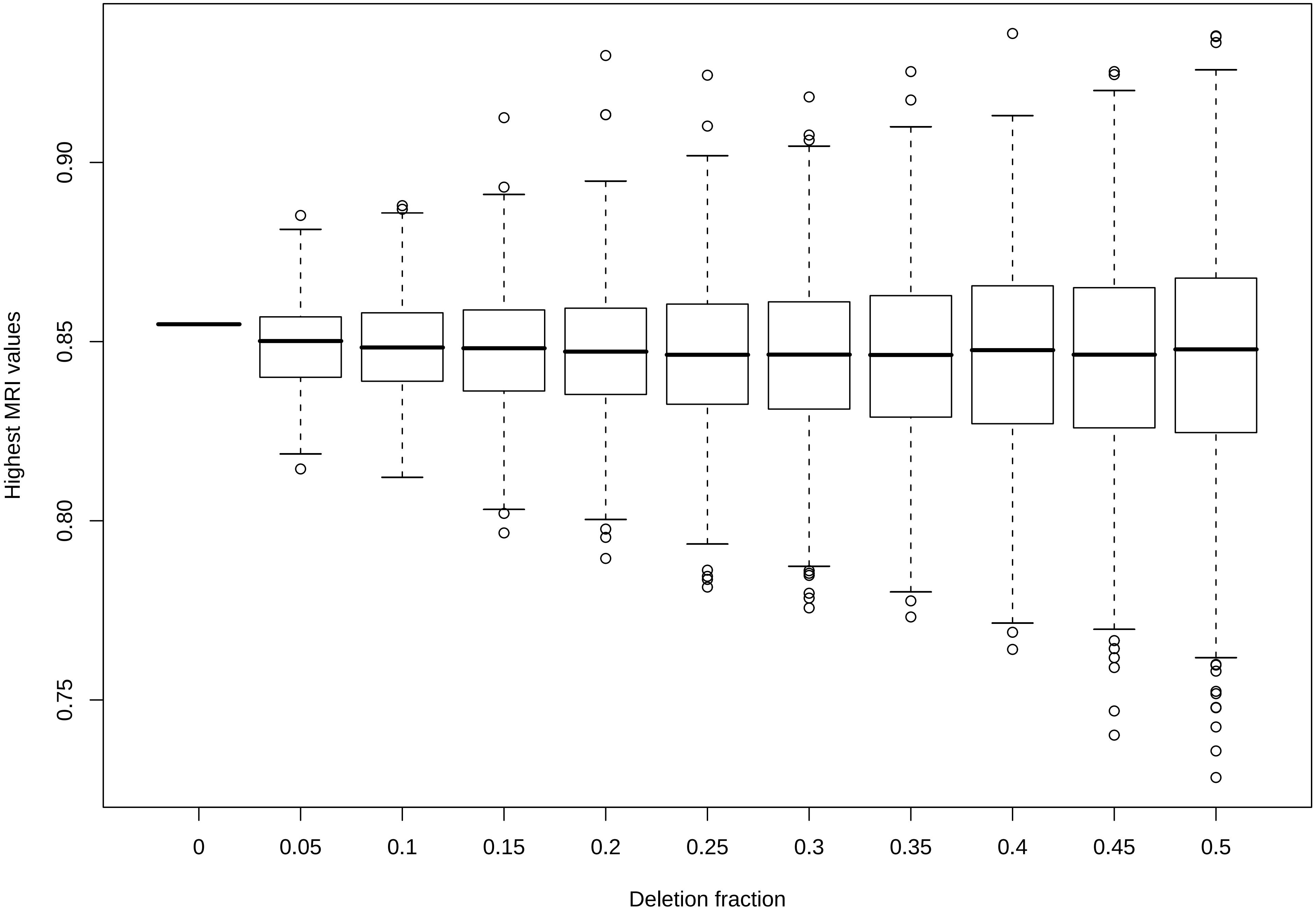
Highest MRI values
0.75
0.80
0.85
0.90
0
0.05
0.1
0.15
0.2
0.25
0.3
0.35
0.4
0.45
0.5
Deletion fraction

Figure 6

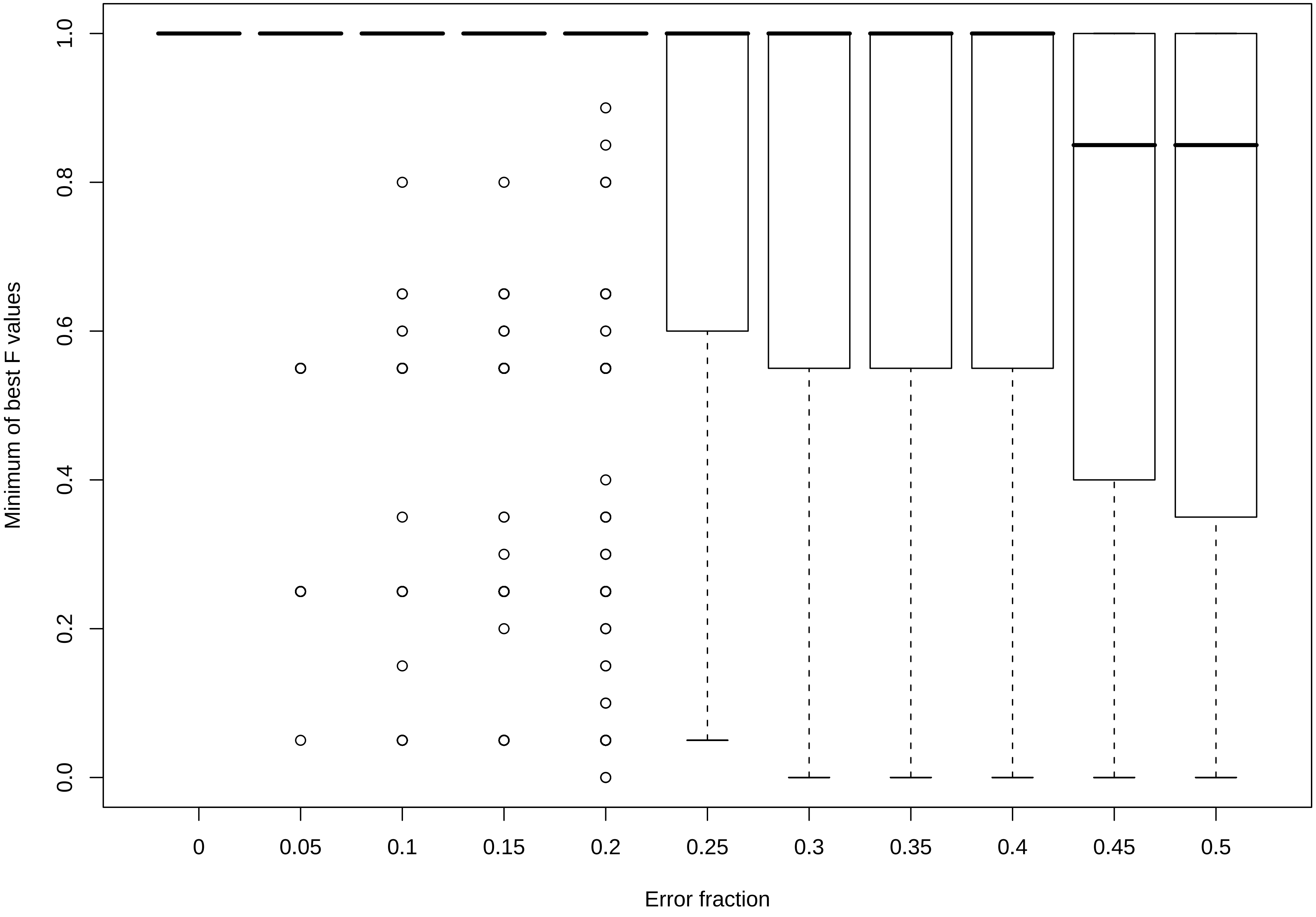
1.0
0.8
0.6
0.4
0.2
0.0
Minimum of best F values
0
0.05
0.1
0.15
0.2
0.25
0.3
0.35
0.4
0.45
0.5
Error fraction

Figure 7

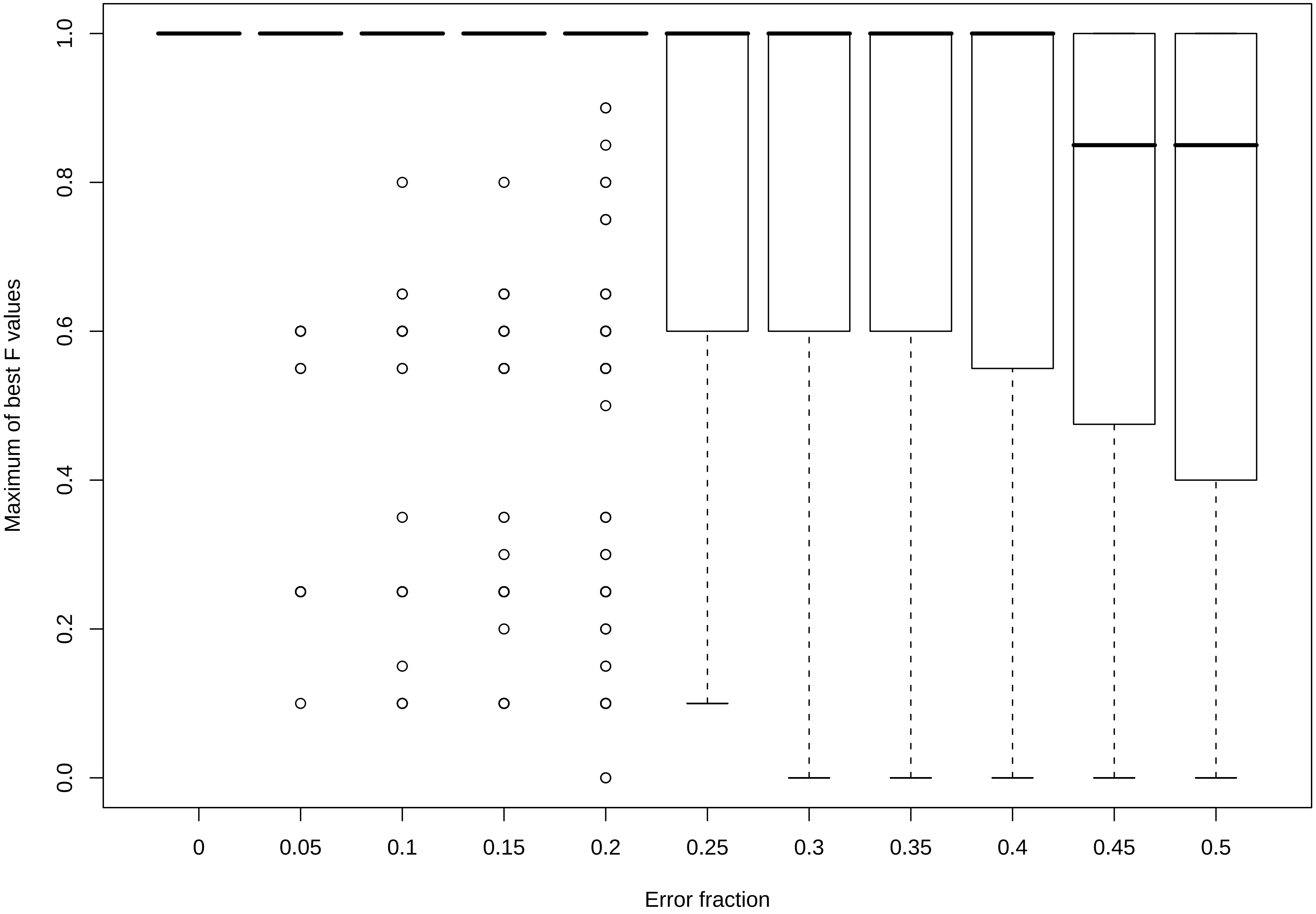
Maximum of best F values
0.0
0.2
0.4
0.6
0.8
1.0
0
0.05
0.1
0.15
0.2
0.25
0.3
0.35
0.4
0.45
0.5
Error fraction

Figure 8

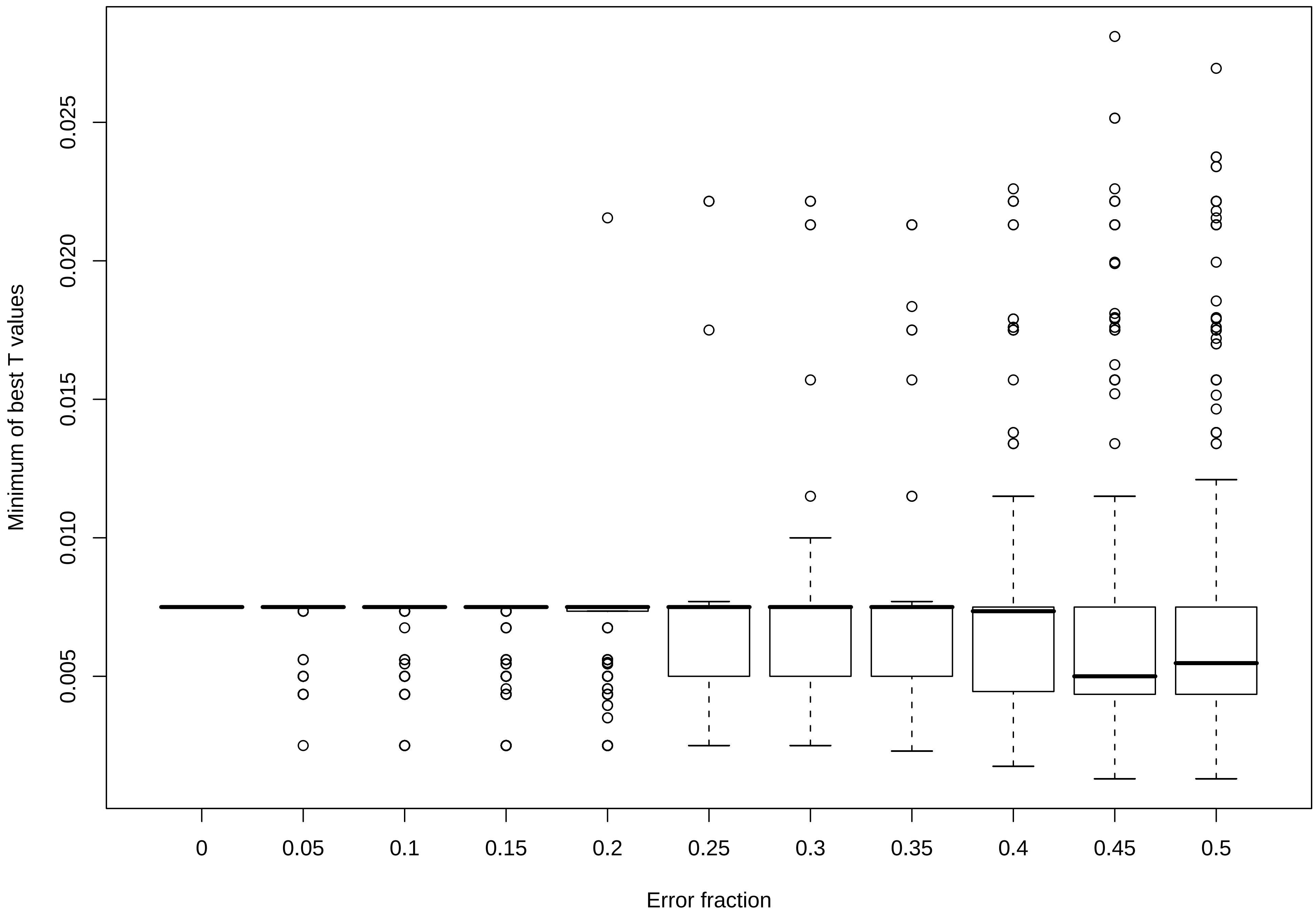

Minimum of best T values
0.005
0.010
0.015
0.020
0.025
0
0.05
0.1
0.15
0.2
0.25
0.3
0.35
0.4
0.45
0.5
Error fraction

Figure 9

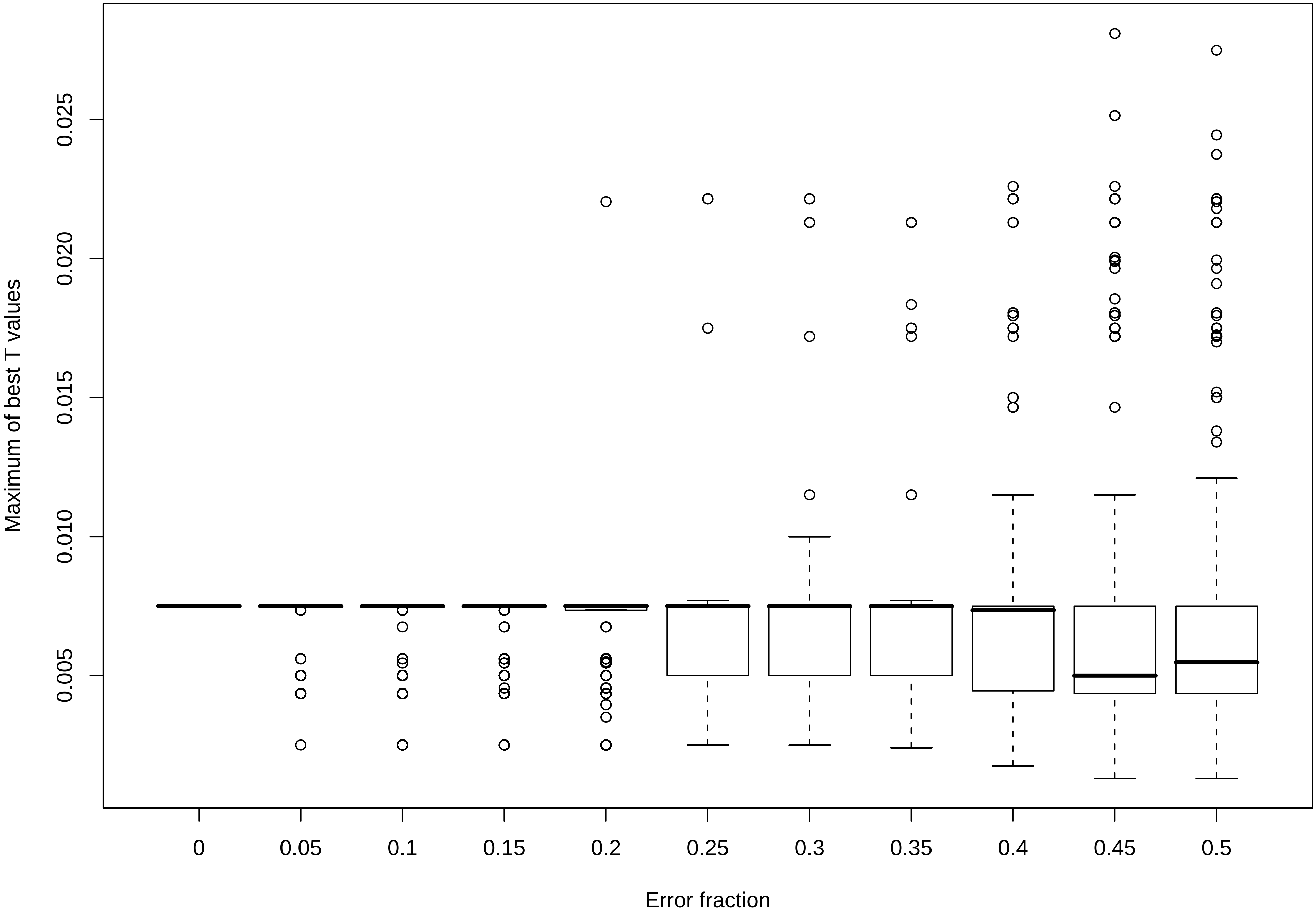

Maximum of best T values
0.005
0.010
0.015
0.020
0.025
0
0.05
0.1
0.15
0.2
0.25
0.3
0.35
0.4
0.45
0.5
Error fraction

Figure 10

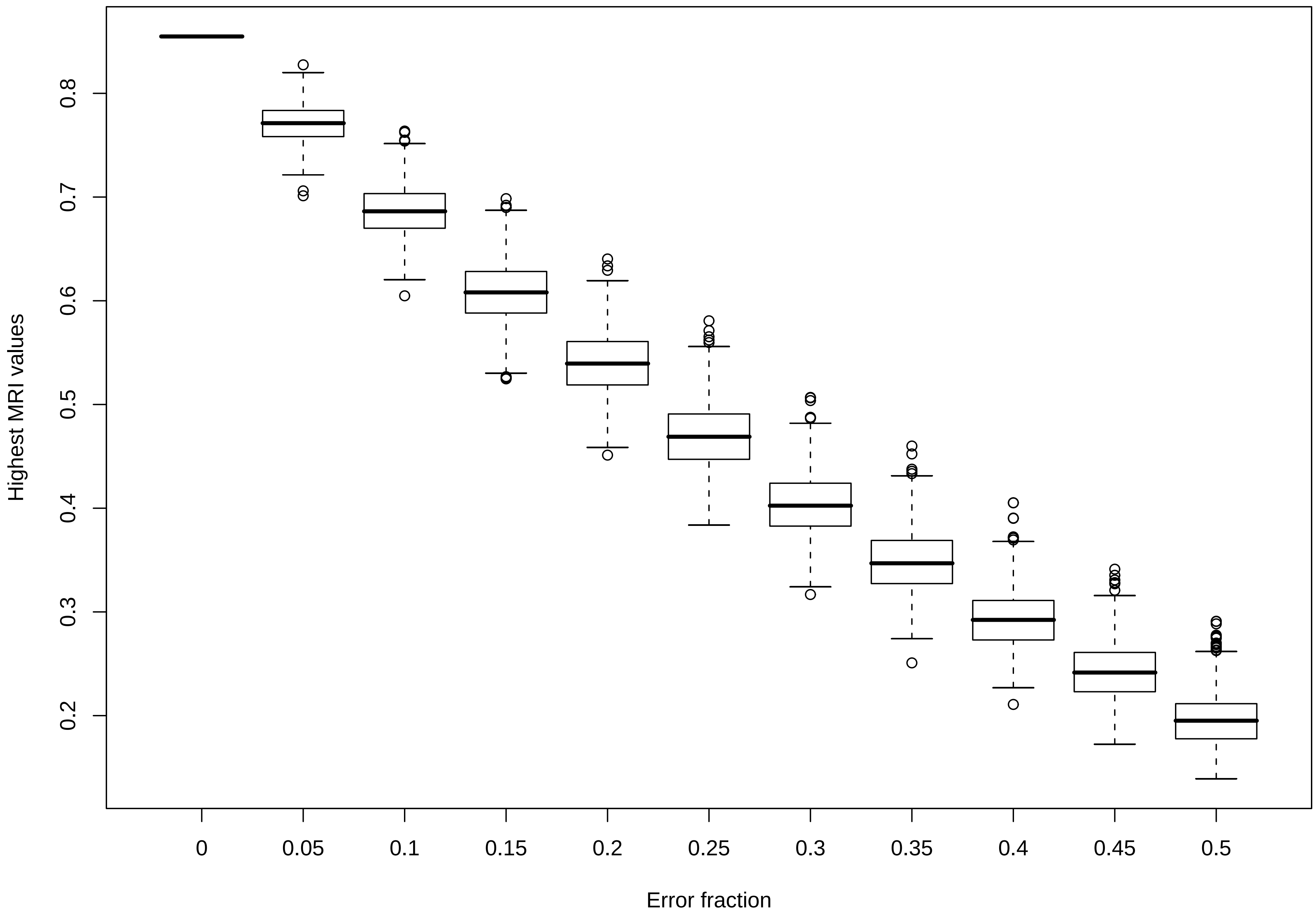
Highest MRI values
0.2
0.3
0.4
0.5
0.6
0.7
0.8
0
0.05
0.1
0.15
0.2
0.25
0.3
0.35
0.4
0.45
0.5
Error fraction

Figure 11

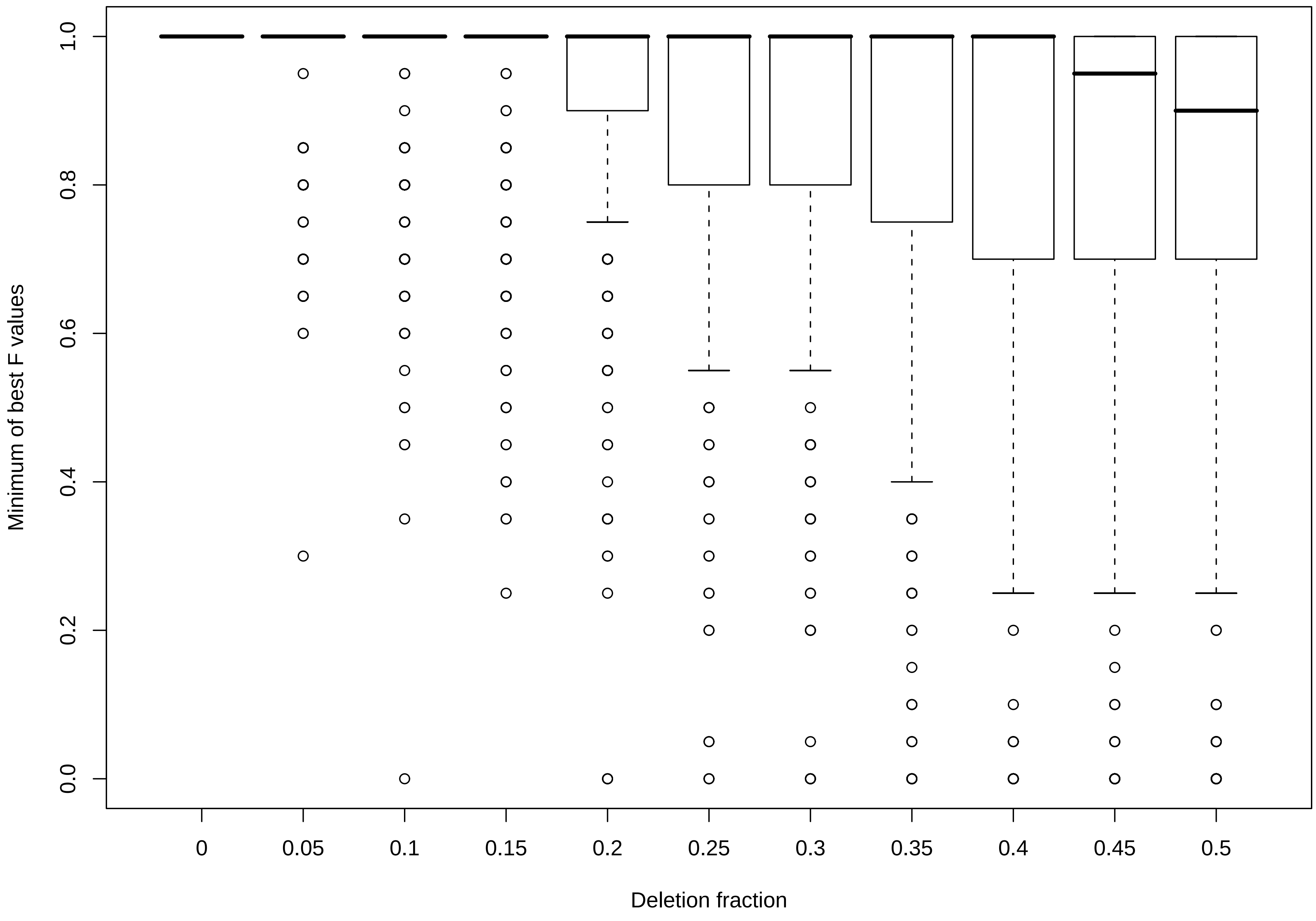

Minimum of best F values
0.0
0.2
0.4
0.6
0.8
1.0
0
0.05
0.1
0.15
0.2
0.25
0.3
0.35
0.4
0.45
0.5
Deletion fraction

Figure 12

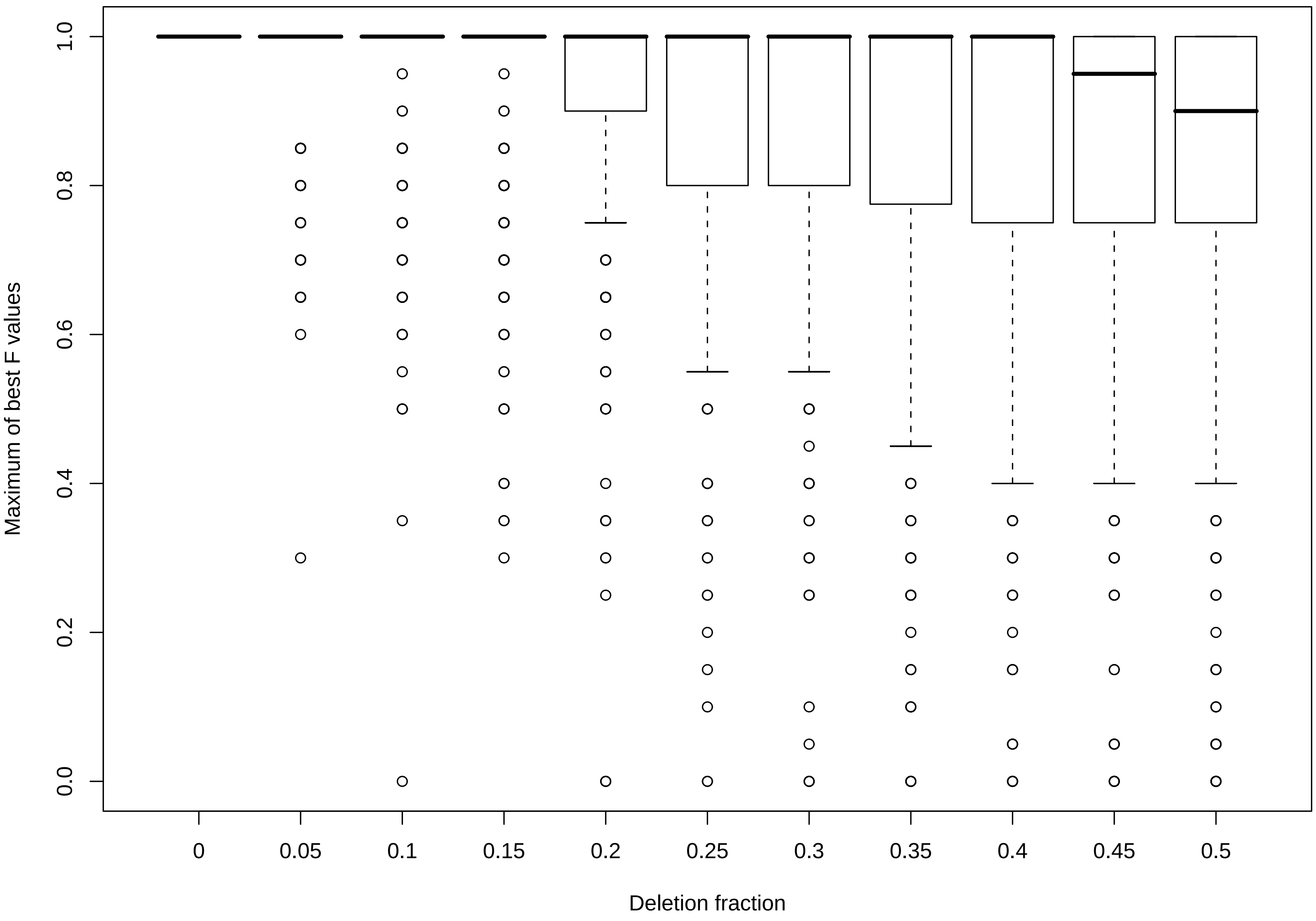

Maximum of best F values
0.0
0.2
0.4
0.6
0.8
1.0
0
0.05
0.1
0.15
0.2
0.25
0.3
0.35
0.4
0.45
0.5
Deletion fraction

Figure 13

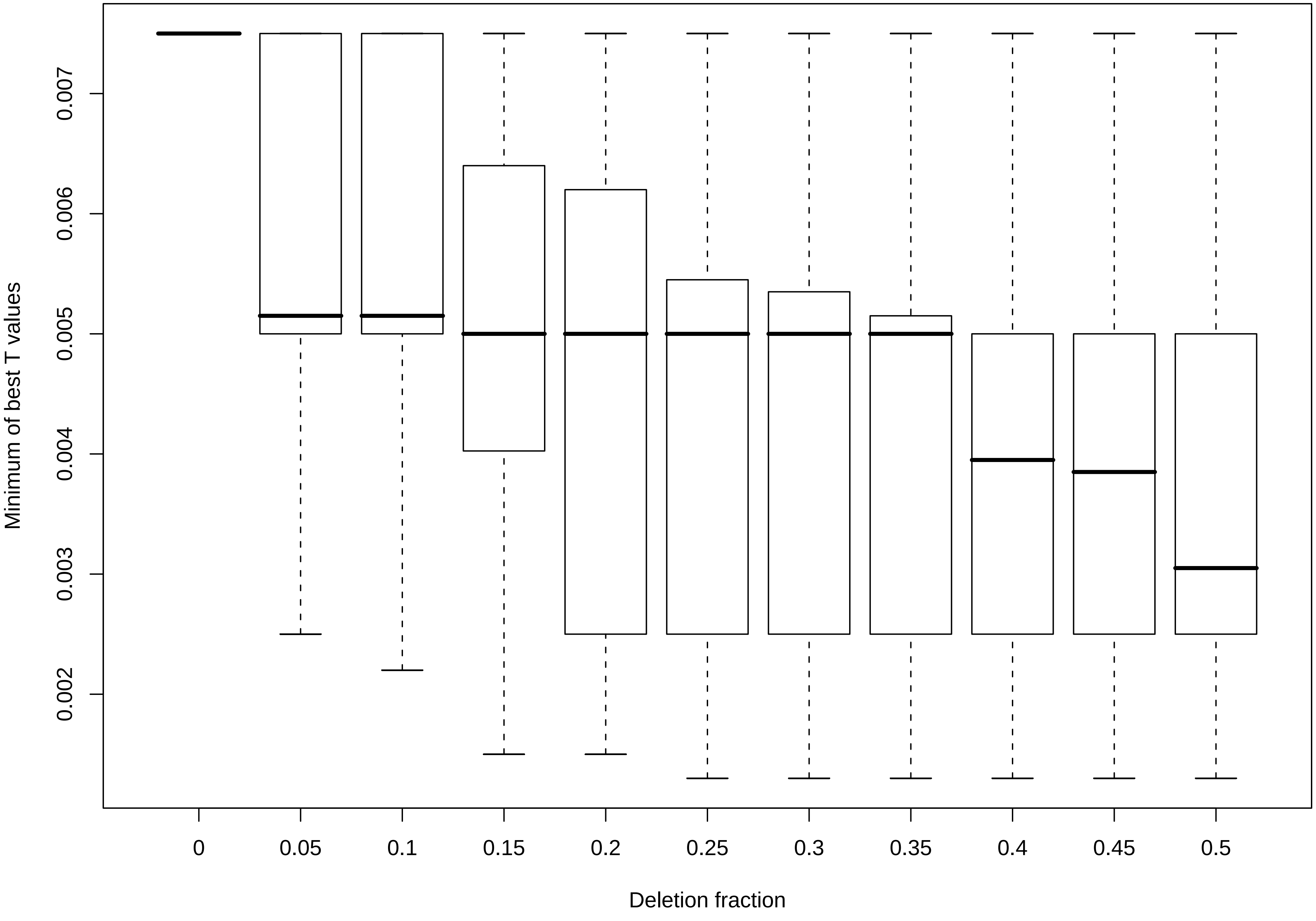

Minimum of best T values
0.002
0.003
0.004
0.005
0.006
0.007
0
0.05
0.1
0.15
0.2
0.25
0.3
0.35
0.4
0.45
0.5
Deletion fraction

Figure 14

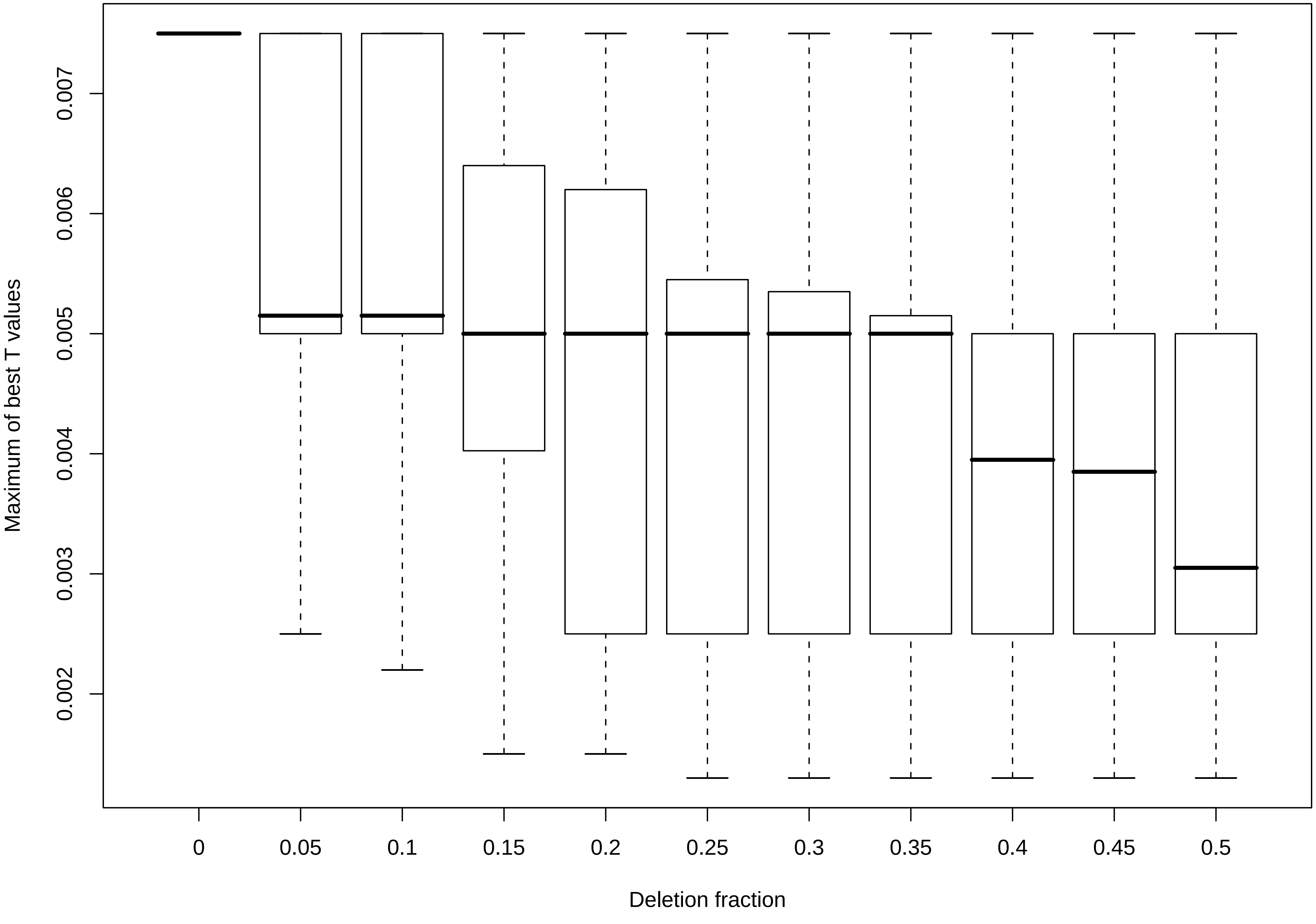

Maximum of best T values
0.002
0.003
0.004
0.005
0.006
0.007
0
0.05
0.1
0.15
0.2
0.25
0.3
0.35
0.4
0.45
0.5
Deletion fraction

Figure 15

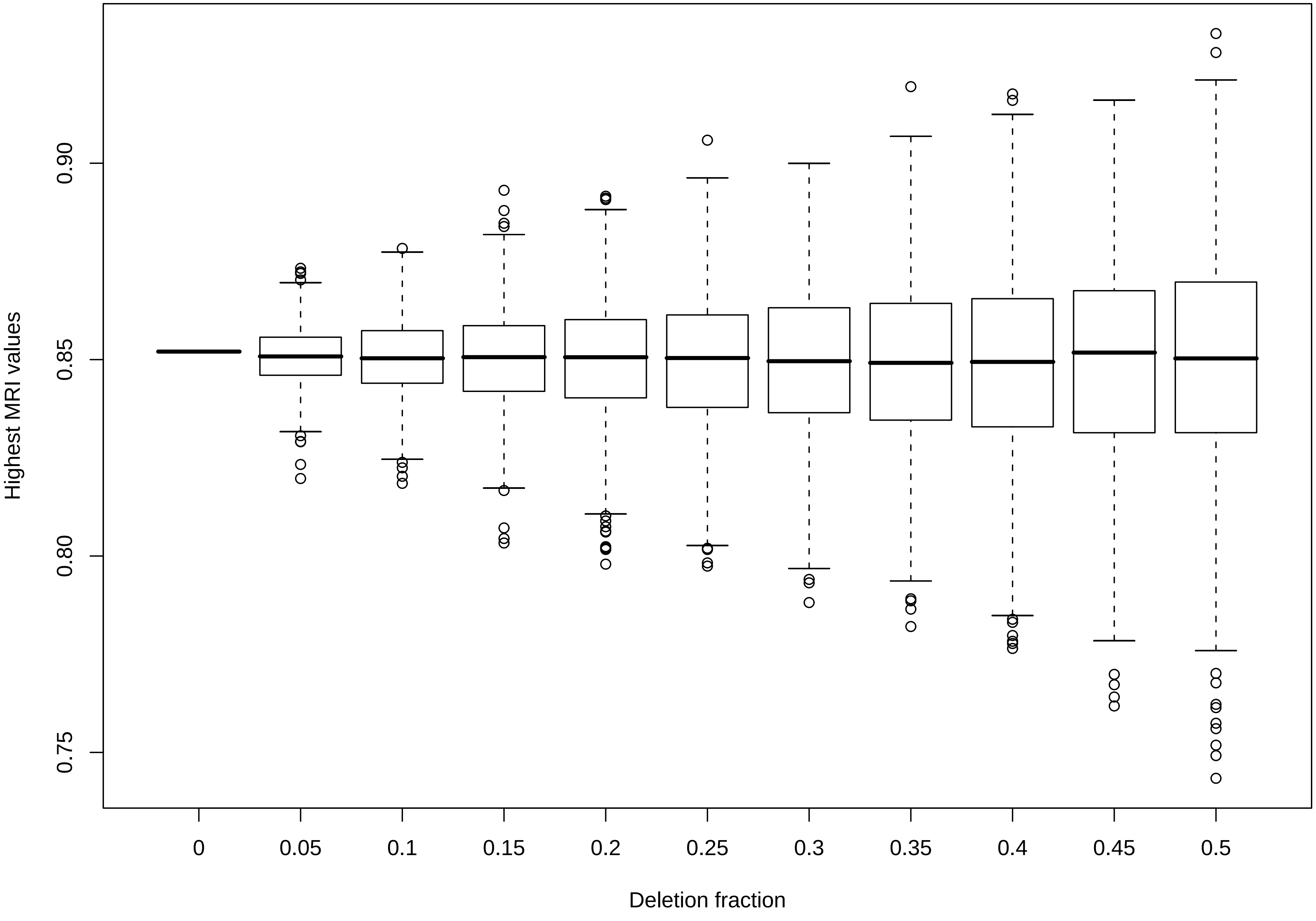

Highest MRI values
0.75
0.80
0.85
0.90
0
0.05
0.1
0.15
0.2
0.25
0.3
0.35
0.4
0.45
0.5
Deletion fraction

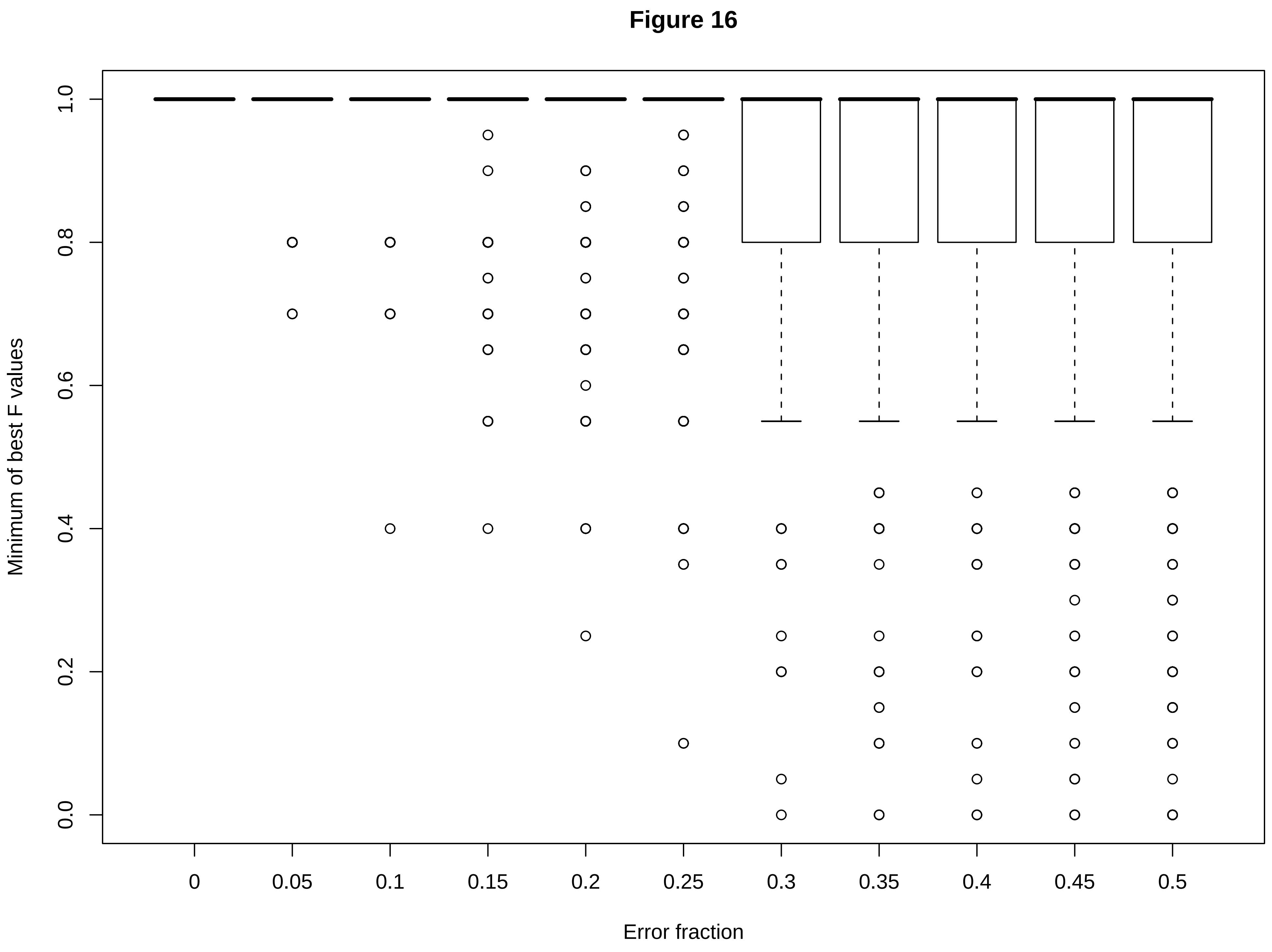
Figure 16
Minimum of best F values
0.0
0.2
0.4
0.6
0.8
1.0
0
0.05
0.1
0.15
0.2
0.25
0.3
0.35
0.4
0.45
0.5
Error fraction

Figure 17

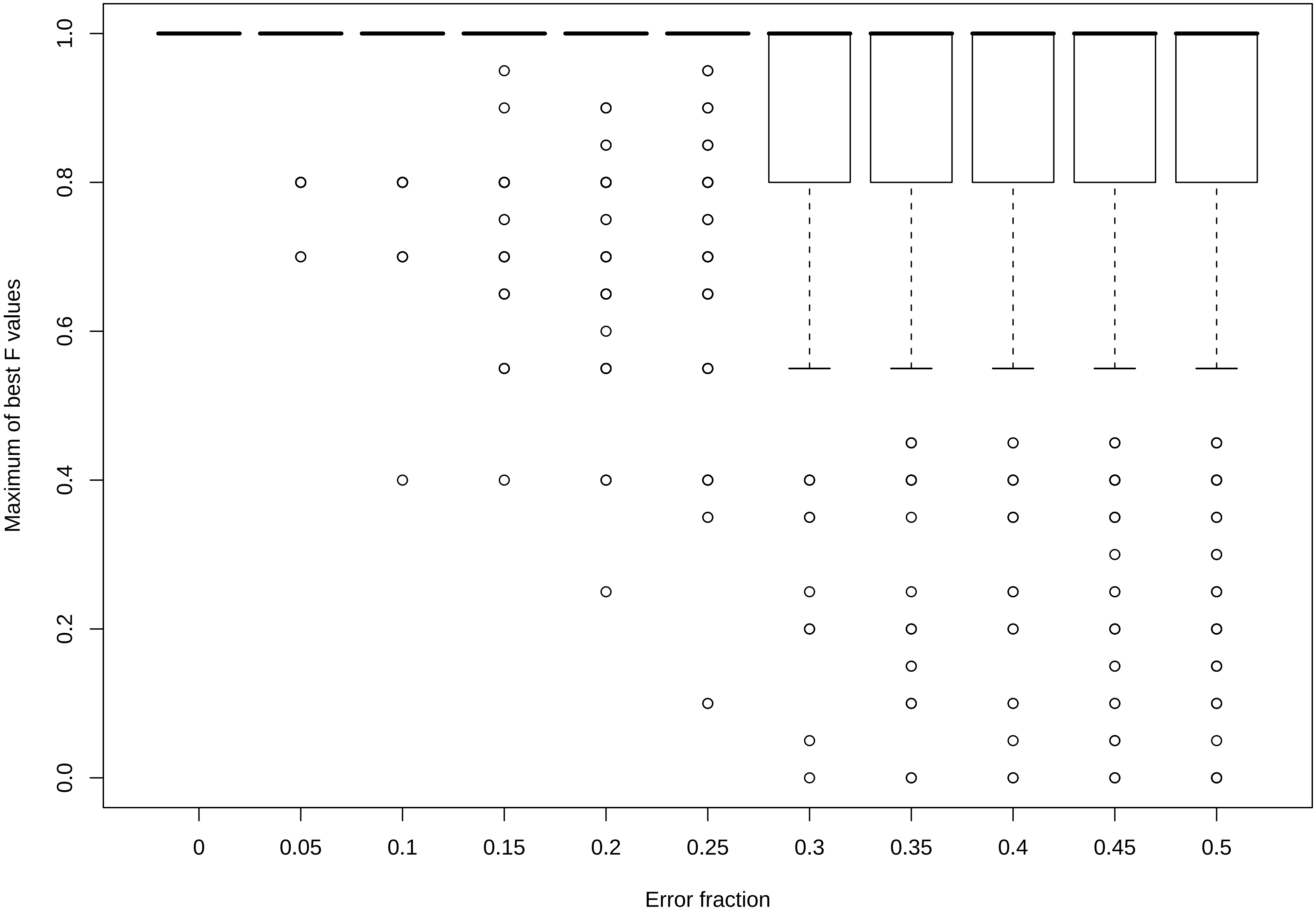

Maximum of best F values
1.0
0.8
0.6
0.4
0.2
0.0
0
0.05
0.1
0.15
0.2
0.25
0.3
0.35
0.4
0.45
0.5
Error fraction

Figure 18

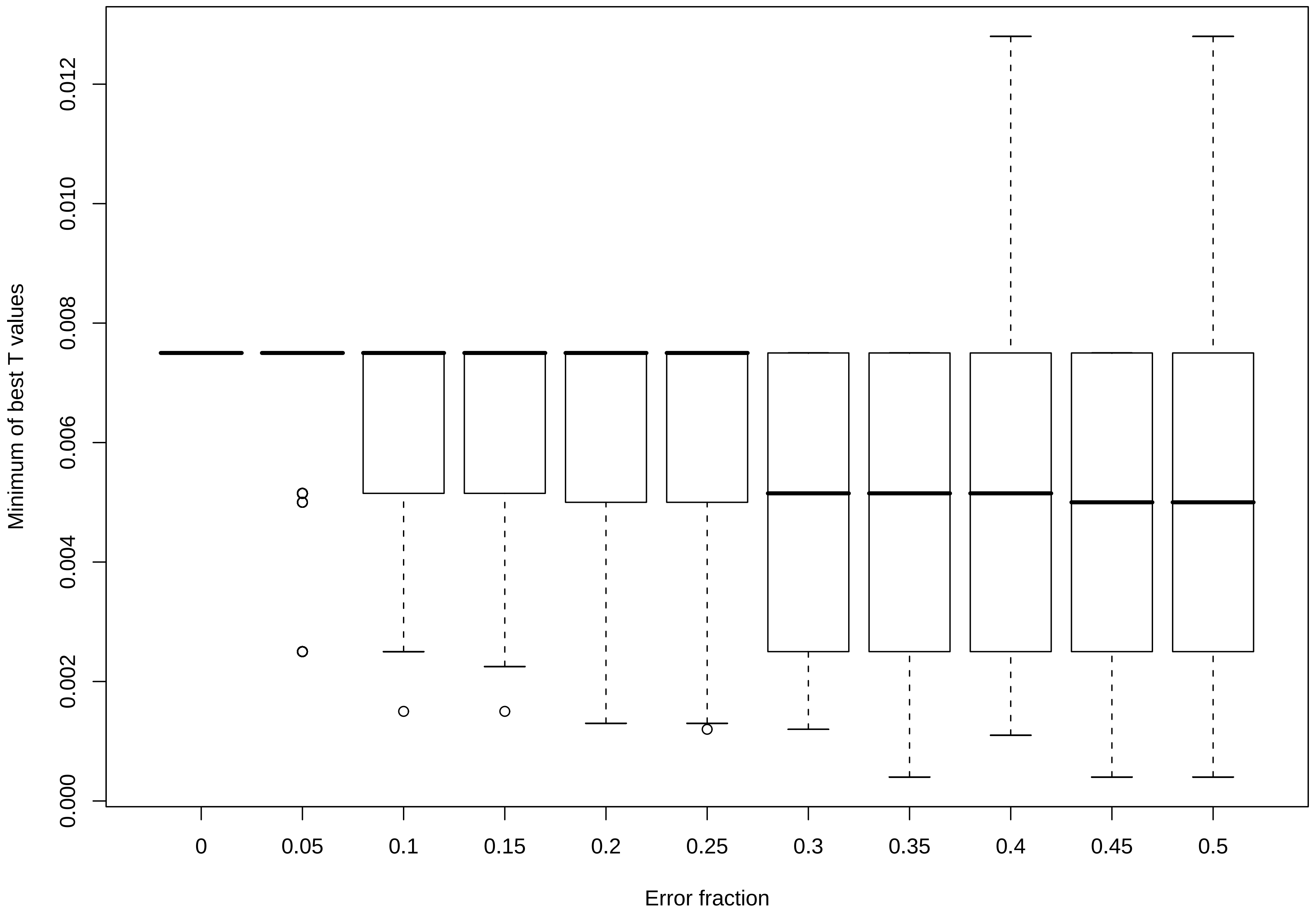

Minimum of best T values
0.000
0.002
0.004
0.006
0.008
0.010
0.012
0
0.05
0.1
0.15
0.2
0.25
0.3
0.35
0.4
0.45
0.5
Error fraction

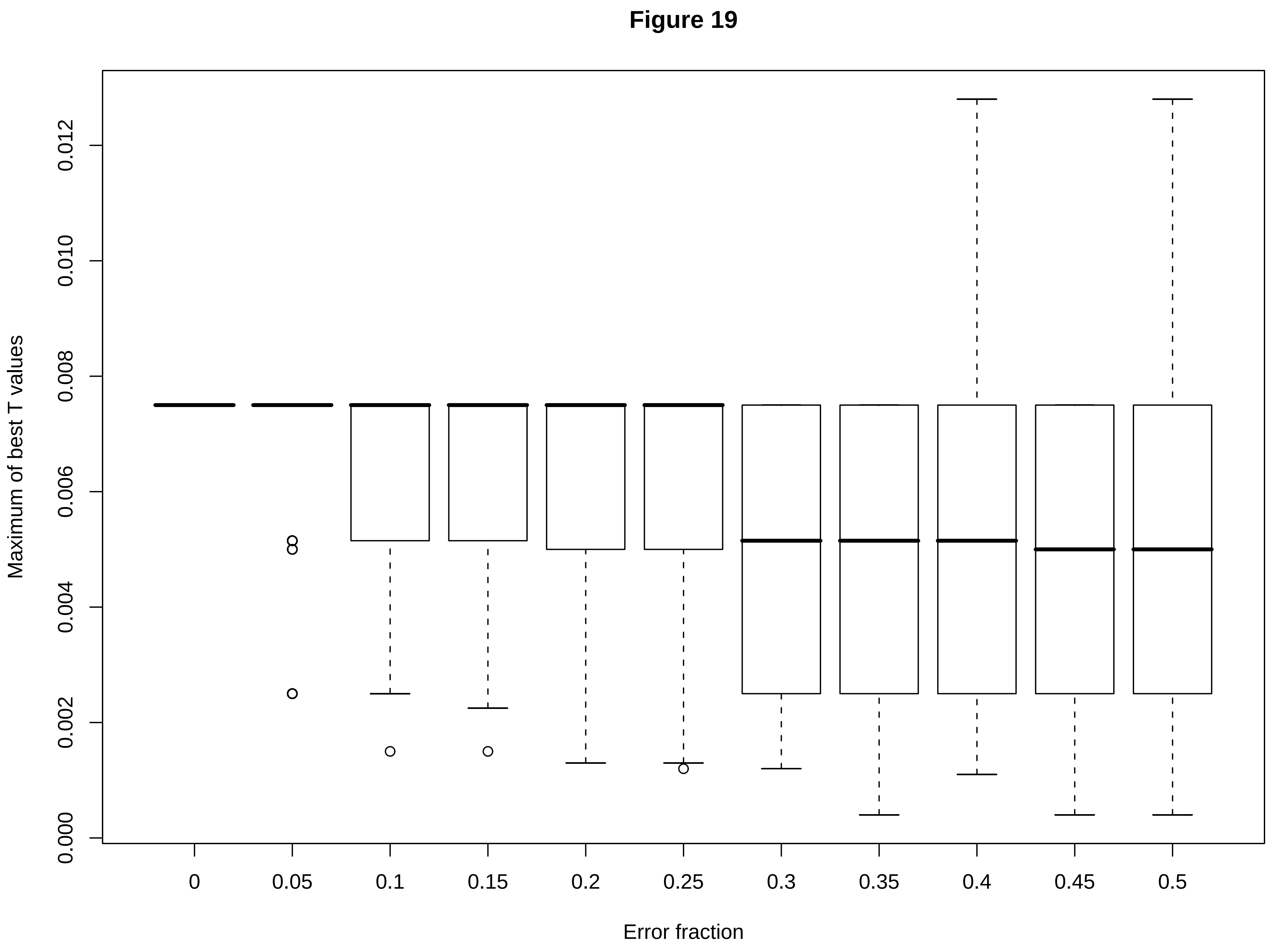
Figure 19
Maximum of best T values
0.000
0.002
0.004
0.006
0.008
0.010
0.012
0
0.05
0.1
0.15
0.2
0.25
0.3
0.35
0.4
0.45
0.5
Error fraction

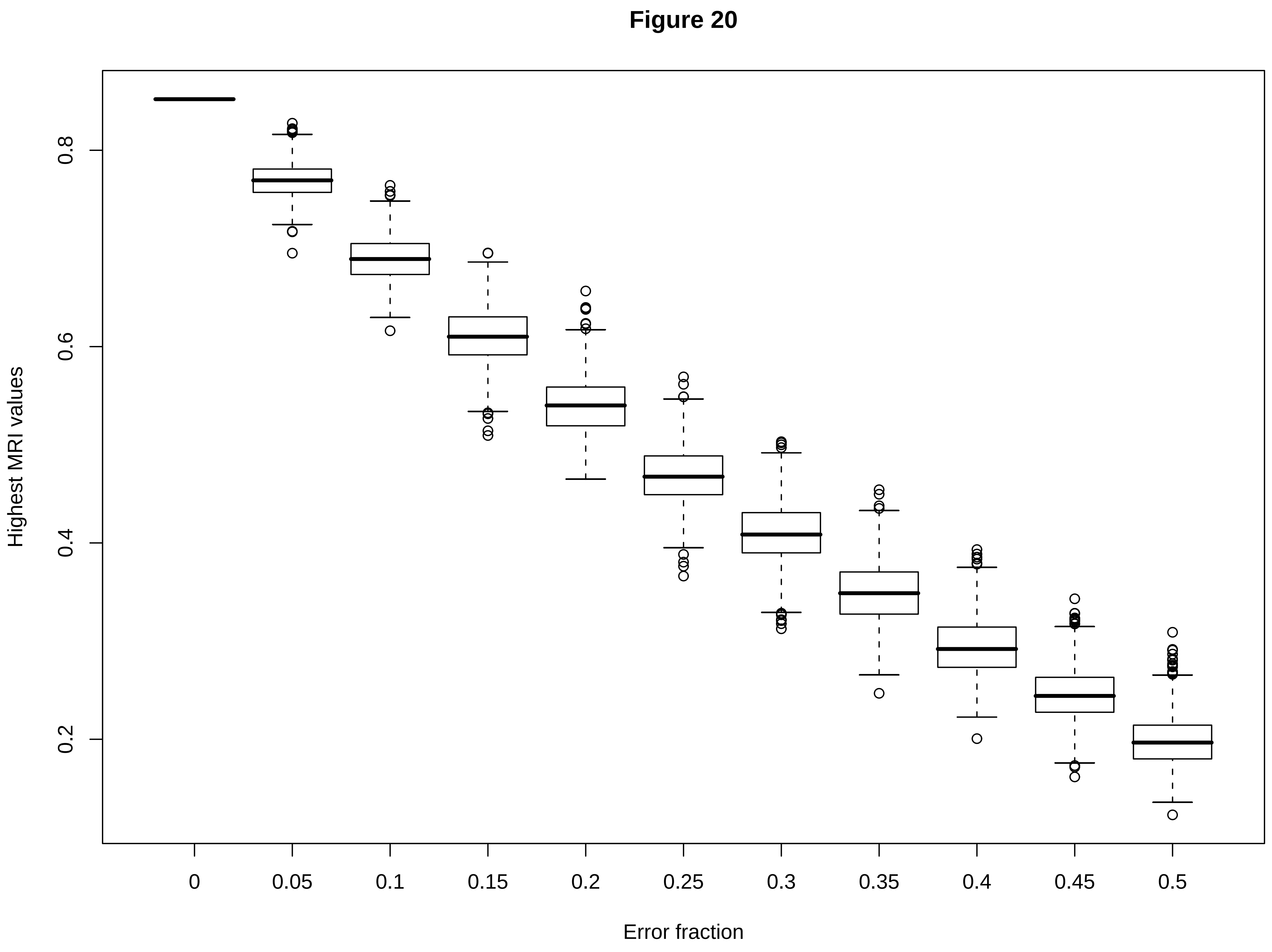
Figure 20
Highest MRI values
0.2
0.4
0.6
0.8
0
0.05
0.1
0.15
0.2
0.25
0.3
0.35
0.4
0.45
0.5
Error fraction
